# Atropisomerism in the Pharmaceutically Relevant Realm

**DOI:** 10.1021/acs.accounts.2c00500

**Published:** 2022-09-26

**Authors:** Mariami Basilaia, Matthew H. Chen, Jim Secka, Jeffrey L. Gustafson

**Affiliations:** Department of Chemistry and Biochemistry, San Diego State University, 5500 Campanile Drive, San Diego, California 92182-1030, United States

## Abstract

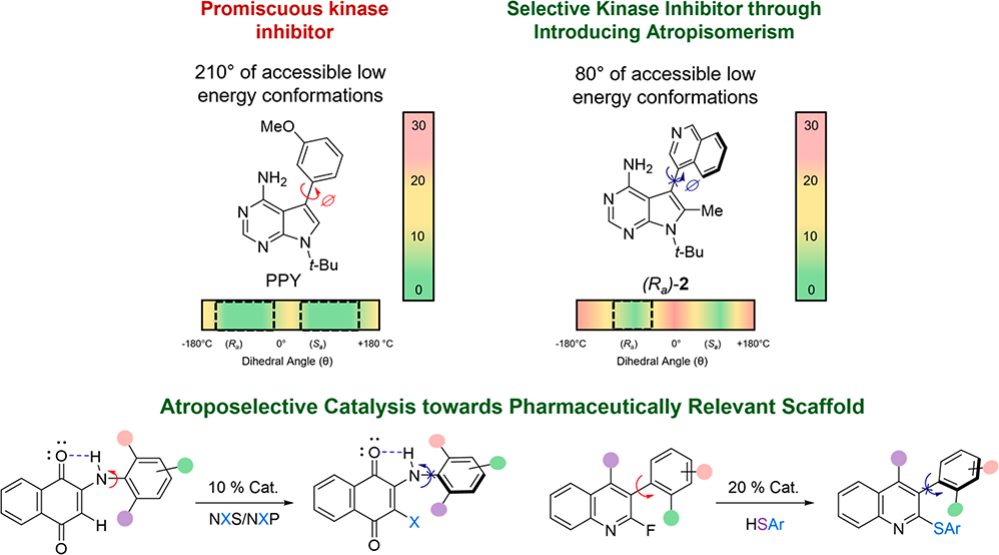

Atropisomerism is a conformational chirality that occurs when there is hindered rotation about a σ-bond. While atropisomerism is exemplified by biaryls, it is observed in many other pharmaceutically relevant scaffolds including heterobiaryls, benzamides, diarylamines, and anilides. As bond rotation leads to racemization, atropisomers span the gamut of stereochemical stability. LaPlante has classified atropisomers based on their half-life of racemization at 37 °C: class 1 (*t*_1/2_ < 60 s), class 2 (60 s < *t*_1/2_ < 4.5 years), and class 3 (*t*_1/2_ > 4.5 years). In general, class-3 atropisomers are considered to be suitable for drug development. There are currently four FDA-approved drugs that exist as stable atropisomers, and many others are in clinical trials or have recently appeared in the drug discovery literature. Class-1 atropisomers are more prevalent, with ∼30% of recent FDA-approved small molecules possessing at least one class-1 axis. While class-1 atropisomers do not possess the requisite stereochemical stability to meet the classical definition of atropisomerism, they often bind a given target in a specific set of chiral conformations.

Over the past decade, our laboratory has embarked on a research program aimed at leveraging atropisomerism as a design feature to improve the target selectivity of promiscuous lead compounds. Our studies initially focused on introducing class-3 atropisomerism into promiscuous kinase inhibitors, resulting in a proof of principle in which the different atropisomers of a compound can have different selectivity profiles with potentially improved target selectivity. This inspired a careful analysis of the binding conformations of diverse ligands bound to different target proteins, resulting in the realization that the sampled dihedral conformations about a prospective atropisomeric axis played a key role in target binding and that preorganizing the prospective atropisomeric axis into a desired target’s preferred conformational range can lead to large gains in target selectivity.

As atropisomerism is becoming more prevalent in modern drug discovery, there is an increasing need for strategies for atropisomerically pure samples of pharmaceutical compounds. This has led us and other groups to develop catalytic atroposelective methodologies toward pharmaceutically privileged scaffolds. Our laboratory has contributed examples of atroposelective methodologies toward heterobiaryl systems while also exploring the chirality of less-studied atropisomers such as diarylamines and related scaffolds.

This Account will detail recent encounters with atropisomerism in medicinal chemistry and how atropisomerism has transitioned from a “lurking menace” into a leverageable design strategy in order to modulate various properties of biologically active small molecules. This Account will also discuss recent advances in atroposelective synthesis, with a focus on methodologies toward pharmaceutically privileged scaffolds. We predict that a better understanding of the effects of conformational restriction about a prospective atropisomeric axis on target binding will empower chemists to rapidly “program” the selectivity of a lead molecule toward a desired target.

## Key References

- SmithD. E.; MarquezI.; LokensgardM. E.; RheingoldA. L.; HechtD. A.; GustafsonJ. L.Exploiting Atropisomerism to Increase the Target Selectivity of Kinase Inhibitors. Angew. Chem., Int. Ed. Engl.2015, 54, 11754–1175910.1002/anie.20150608526276764.^1^*Initial proof of concept that class-3 atropoisomerism can be introduced into rapidly interconverting class-1 atropisomeric kinase inhibitors to modulate target selectivity.*
- ToenjesS. T.; GustafsonJ. L.Atropisomerism in Medicinal Chemistry : Challenges and Opportunities. Future Med. Chem.2018, 10, 409–42210.4155/fmc-2017-015229380622PMC5967358.^2^*An overview of atropisomerism in drug discovery that includes an analysis of recent FDA-approved drugs that found ∼one-third possessed at least one potential axis of atropisomerism*.
- ToenjesS. T.; GarciaV.; MaddoxS. M.; DawsonG. A.; OrtizM. A.; PiedrafitaF. J.; GustafsonJ. L.Leveraging Atropisomerism to Obtain a Selective Inhibitor of RET Kinase with Secondary Activities toward EGFR Mutants. ACS Chem. Biol.2019, 14, 1930–193910.1021/acschembio.9b0040731424197PMC7259470.^3^*Atropisomerism was leveraged to obtain a selective RET inhibitor. The authors analyzed the conformations of ∼110 similar ligands bound to kinases in the PDB and found that RET selectivity was driven by preorganizing the axis into “RET optimal”conformations.*
- VaidyaS. D.; ToenjesS. T.; YamamotoN.; MaddoxS. M.; GustafsonJ. L.Catalytic Atroposelective Synthesis of N-Aryl Quinoid Compounds. J. Am. Chem. Soc.2020, 142, 2198–220310.1021/jacs.9b1299431944689PMC7239344.^4^*Study in which intramolecular hydrogen bonding was leveraged to obtain class-3 atropisomeric N-arylquinoids, a scaffold related to diarylamines. These scaffolds were prepared in a catalytic atroposelective fashion via a chiral phosphoric acid-catalyzed bromination*.

## 1 Introduction

**Figure 1:**
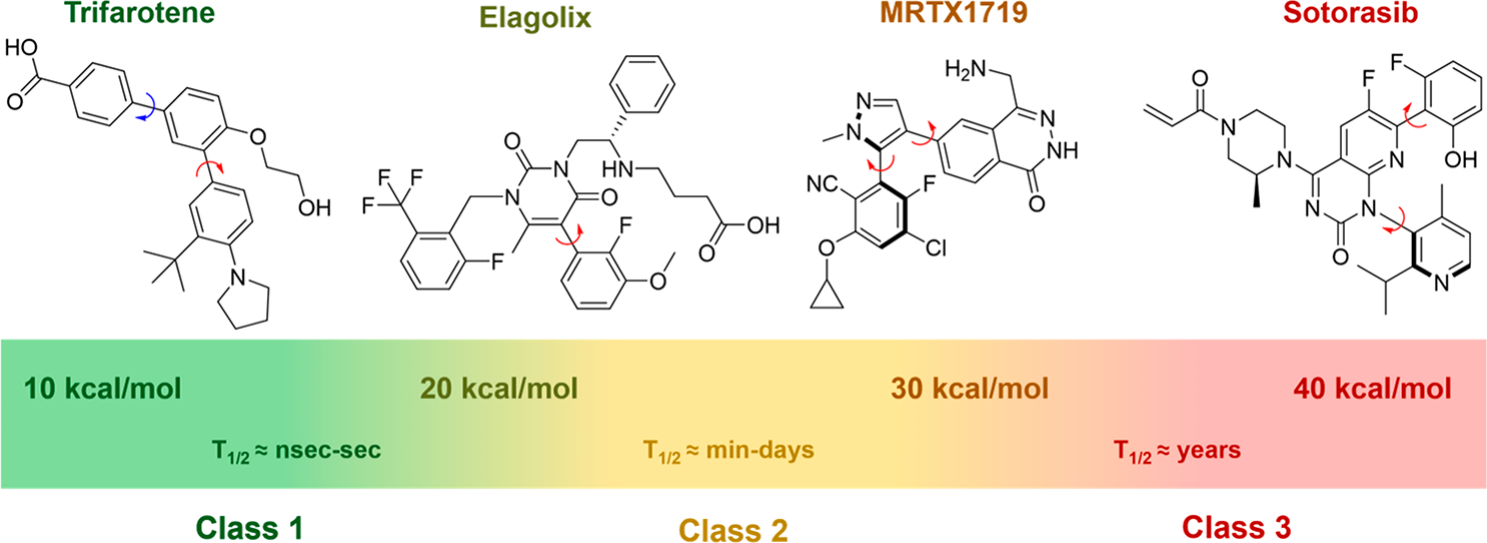
Spectrum of stereochemical stability for atropisomers. Atropisomeric axes are denoted by red arrows. Pro-atropisomeric axes are denoted by blue arrows.

Atropisomerism, which was first observed a century ago,^5^ is a type of axial chirality that arises when there is hindered rotation about a bond. The term atropisomer is derived from the Greek word “atropos” meaning “without turn”.^6^ Atropisomerism can be thought of as a dynamic form of chirality as bond rotation represents a spontaneous mechanism of racemization. However, as the name suggests, the arbitrary definition of atropisomers is conformers that do not readily interconvert, with the classical standard being those with a half-life of interconversion of >1000 s at a given temperature. A decade ago, LaPlante^7,8^ classified atropisomers based on their half-life of racemization at 37 °C: class 1 (*t*_1/2_ < 60 s), class 2 (60 s < *t*_1/2_ > 4.5 years), and class 3 (*t*_1/2_ > 4.5 years; corresponding Δ*G*^⧧^ values of racemization are included in Figure 1). Class-1 atropisomers do not meet the classical definition of atropisomerism and are treated as achiral, while class-3 atropisomers are treated as stable enantiomers. Class-2 atropisomers, which can be observed by NMR and even isolated in many cases, racemize on the minute to month time scale and have been referred to as “a lurking menace”^9^ due to regulatory-based complications that are caused by the lack of stereochemical stability.

Atropisomerism has become increasingly prevalent in modern drug discovery over the past decade. There have been four FDA-approved class-3 atropisomers: telenzepine^10^ (administered as a racemate), colchicine (which also possesses a point chiral center and primarily exists as a single diastereoisomer),^11^ lesinurad (which has been discontinued),^12^ and sotorasib.^13^ A recent analysis from our group^2^ found that ∼30% of recent FDA-approved small molecules (2010–2018) possess at least one class-1 atropisomeric axis. The increasing prevalence of atropisomerism of all classes of stability in drug discovery can perhaps be explained by the rise of aromatic heterocyles as common functional groups that positively contribute to the various drug properties (i.e., potency via interactions with target protein, ADME, and PK) that are important in drug development. This is also underscored by the reactions commonly employed in early-stage drug discovery,^14^ with reaction classes such as amide couplings (benzamides),^15,16^ cross-couplings (biaryls and heterobiaryls),^17^ nucleophilic aromatic substitution (S_N_Ar, diarylamines),^18,19^ and electrophillic aromatic substitution (S_E_Ar) all being common reaction types employed on aromatic heterocycles capable of yielding atropisomeric scaffolds.^4,15,20−22^ While much has been written about how the prevalence of aromatics in drug discovery has led to flat molecules that sample little chemical space,^23^ our group has shown that class-1 atropisomeric axes are anything but “flatland” as (1) they can sample the full 360° of rotational conformations about the axis; (2) they bind a given target in only a small subset of these conformations; and (3) different targets can prefer different subsets of conformations about the same axis.

Obtaining selective small-molecule inhibitors is one of the most challenging aspects of drug discovery and is exceedingly important, as off-target inhibition can lead to adverse events in patients and failure in the clinic. Often the pursuit of selectivity will result in drawn-out optimization studies that can lead to compounds that are at the periphery of “drug-likeness” that may now possess other liabilities. As such, there is a need for generalizable strategies that allow for the systematic modulation of the target selectivity of lead compounds. The ubiquity of prospective atropisomerism in drug discovery led us to explore the potential of leveraging atropisomerism as a design element to modulate the target selectivity of biologically active small molecules. As we embarked on this work, we became aware of a lack of enantioselective methodologies toward many classes of pharmaceutically relevant atropisomer, leading us to explore general strategies toward the atroposelective synthesis of these motifs. In this Account, we aim to offer a succinct overview of atropisomerism in drug discovery as well as our work on leveraging atropisomerism to obtain more selective small molecules and as an inspiration for new chemistry.

## 2 Recent Examples of Atropisomerism in Drug Discovery

**Figure 2:**
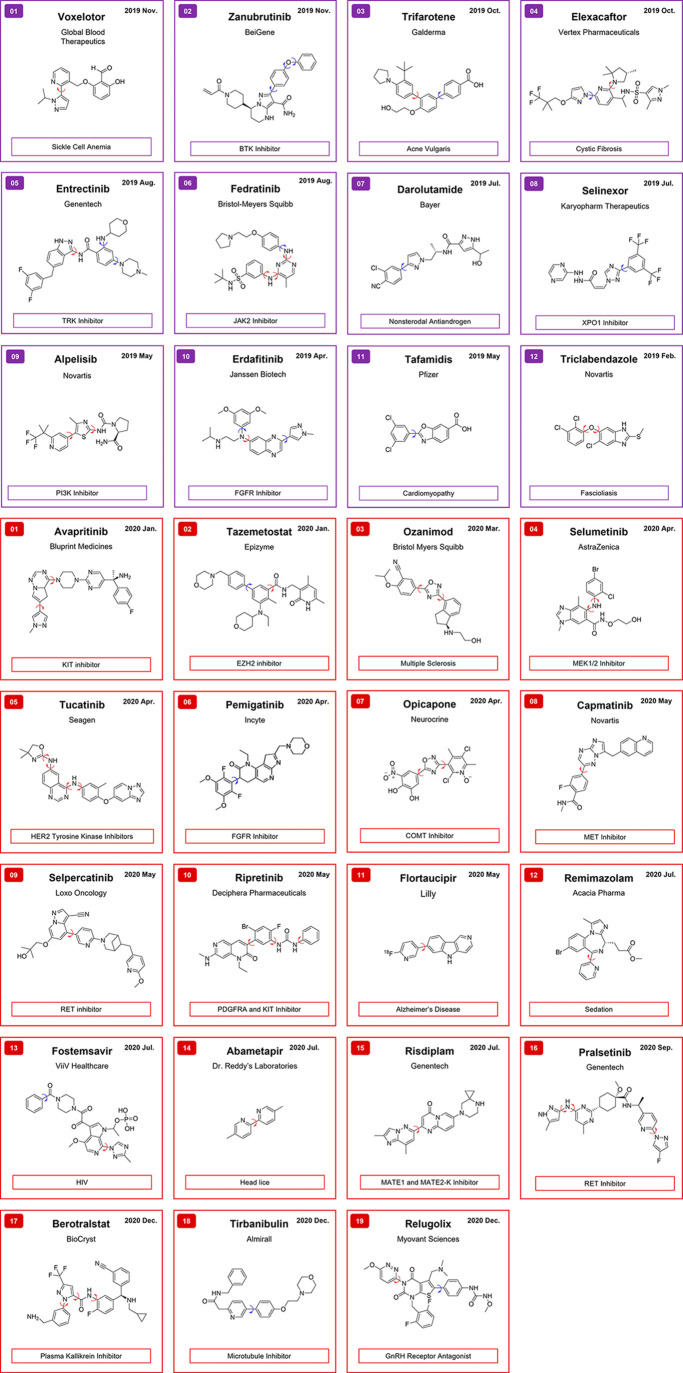

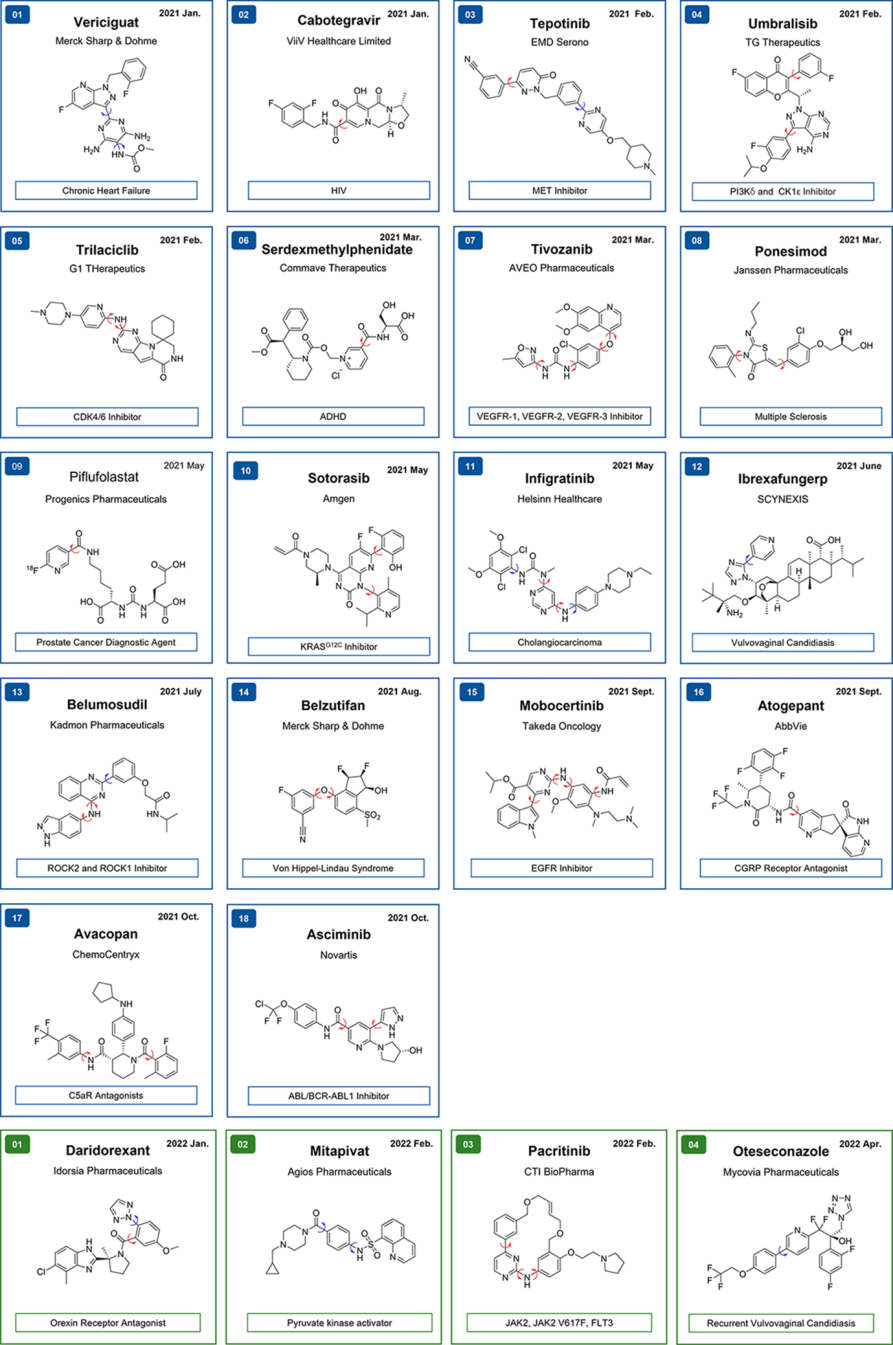
Examples of FDA approved drugs that possess a prospective atropisomeric axis. Atropisomeric axes are denoted by red arrows. Pro-atropisomeric axes are denoted by blue arrows. 2019–2022 approvals are color-coded by year (2019, purple; 2020, red; 2021, blue; and 2022, green).

**Figure 3:**
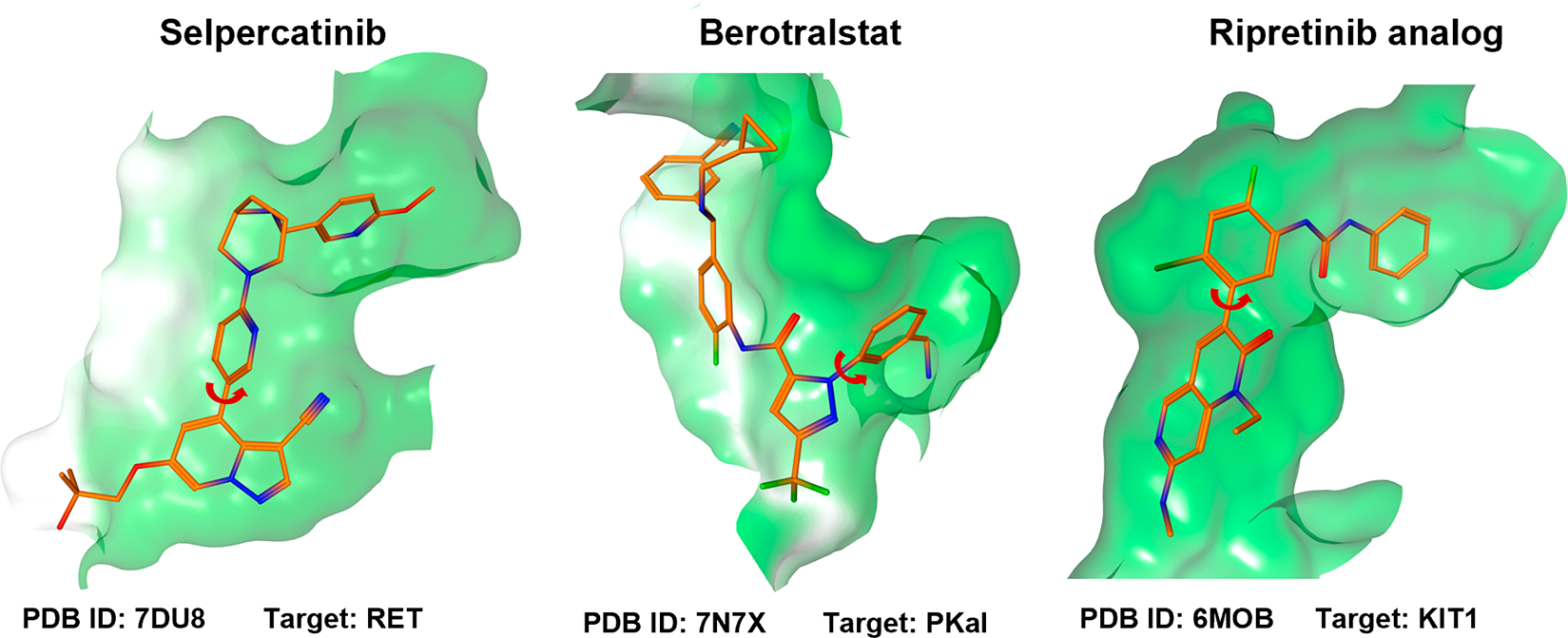
Co-crystal structures of FDA-approved class-1 atropisomers bound to a target in the single atropisomeric conformation.

Scaffolds that can potentially exhibit atropisomerism are common among the privileged motifs in modern drug discovery. Between 2019 and early 2022, there have been 43 FDA-approved small molecules that possess an atropisomeric axis (Figure 2) of any of LaPlante’s classes of atropisomer stability, representing 26% of all small-molecule approvals over that time. Another 10 drugs possess a symmetrical “pro-atropisomeric axis” (denoted by the blue arrow). A majority of these examples exist as class-1 atropisomers at 37 °C. Analyses of data in the Protein Data Bank (PDB) reveal that a majority of these molecules (i.e., selpercatinib, ripretinib, and berostralstat; see Figure 3) bind their given target in a single set of chiral conformations. Elagolix,^24^ which was approved in 2018 for endometriosis, is an example of a recent class-2 atropisomer that has been FDA-approved with a Δ*G*^⧧^ of 23.3 kcal/mol corresponding to an extrapolated *t*_1/2_ of racemization of ∼45 min under physiological conditions. Sotorasib (AMG-510), a first-in-class mutant KRAS G12C inhibitor, represents the most recent class-3 atropisomer to be FDA-approved and was determined to have a Δ*G*^⧧^ of racemization of greater than 31 kcal/mol, with its atropisomer configuration proving key to its medicinal chemical optimization.^13,25^

**Figure 4:**
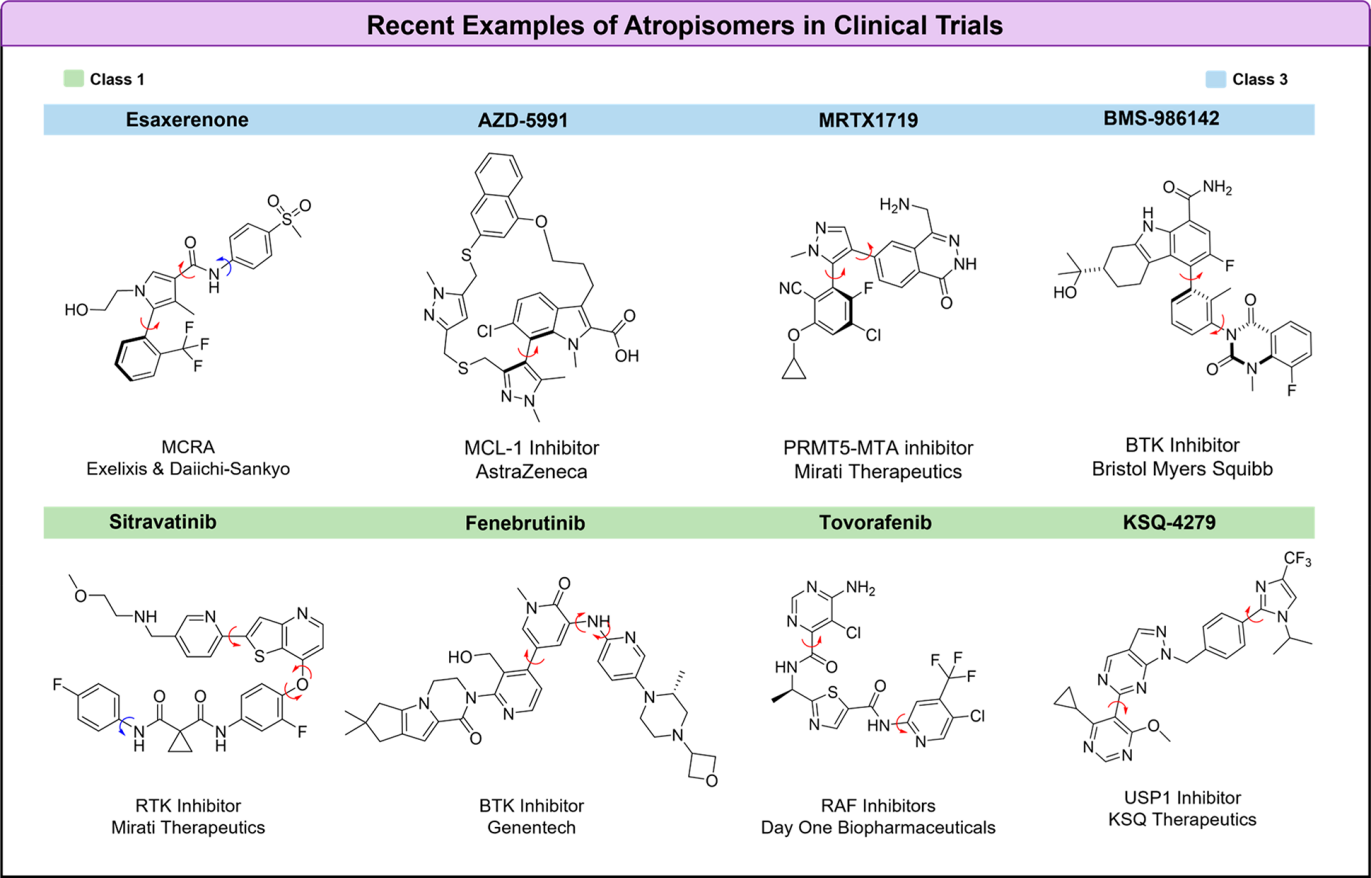
Examples of atropisomers that have undergone recent clinical trials.

There are also several examples of atropisomerism currently in clinical trials (Figure 4). Recent examples of class-3 atropisomers include BMS’s noncovalent BTK inhibitor BMS-986142^26^ (which possesses a class-3 and a class-2 atropisomeric axis), Astra-Zeneca’s MCL-1 inhibitor AZD-5991,^27^ and Mirati’s PRMT5-MTA inhibitor MTRX-1719.^28^ Esaxerenone, a nonsteroidal mineralocorticoid receptor antagonist (MCRA) developed by Daiichi-Sankyo and approved in Japan^29^ for the treatment of hypertension, also exists as isolable atropisomers, with one atropisomer possessing the majority of activity.^29,30^ Unsurprisingly, there are fewer examples of class-2 atropisomers in clinical trials. There are myriad examples of class-1 atropisomers currently in clinical trials, with a few illustrative examples in Figure 4.

**Figure 5:**
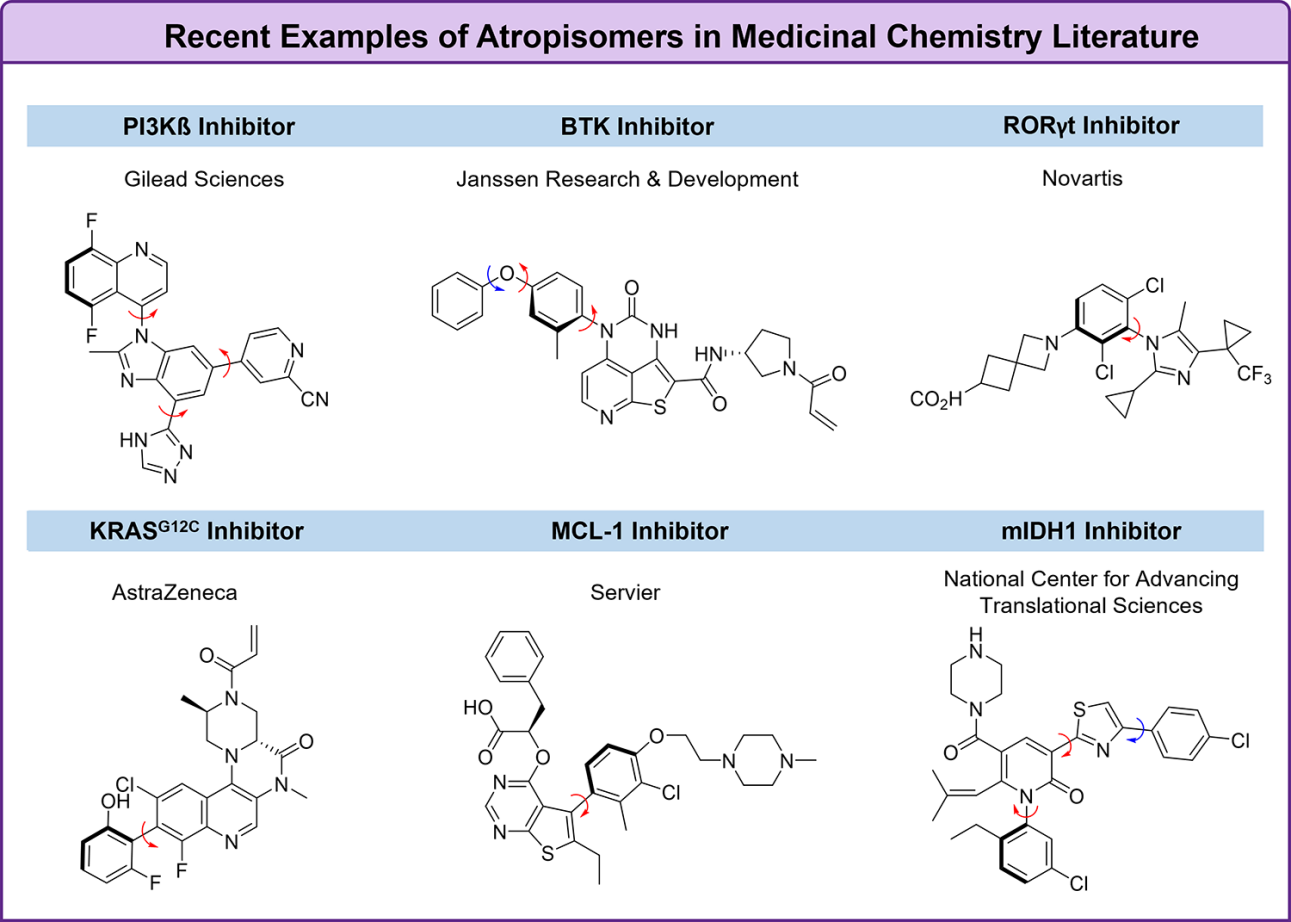
Examples of stable atropisomers from the recent medicinal chemical literature.

There have also been myriad examples of class-3 atropisomers in the recent medicinal chemistry literature (Figure 5). Gilead published a series of papers^31,32^ that led to the discovery of a selective PI3Kβ inhibitor that existed as a class-3 atropisomer. A key finding of this work was the recognition that a lead compound bound the target in a nearly orthogonal conformation, leading them to evaluate class-3 atropisomeric analogs. Janssen, Novartis, and AstraZeneca made similar observations that led to potent and selective inhibitors of BTK,^33^ RORγt,^34^ and KRAS G12C,^35^ respectively. Servier^36^ has disclosed an atropisomeric MCL-1 inhibitor that possesses both a class-3 atropisomeric axis and an instance of point chirality, where the introduction of the class-3 atropoisomeric axis proved to be vital for selectivity. Finally, researchers from NIH disclosed an mIDH1 inhibitor that possessed a class-3 atropisomeric heterobiaryl.^37^

## 3 Leveraging the Atropisomer Conformation to Modulate Target Selectivity

**Figure 6:**
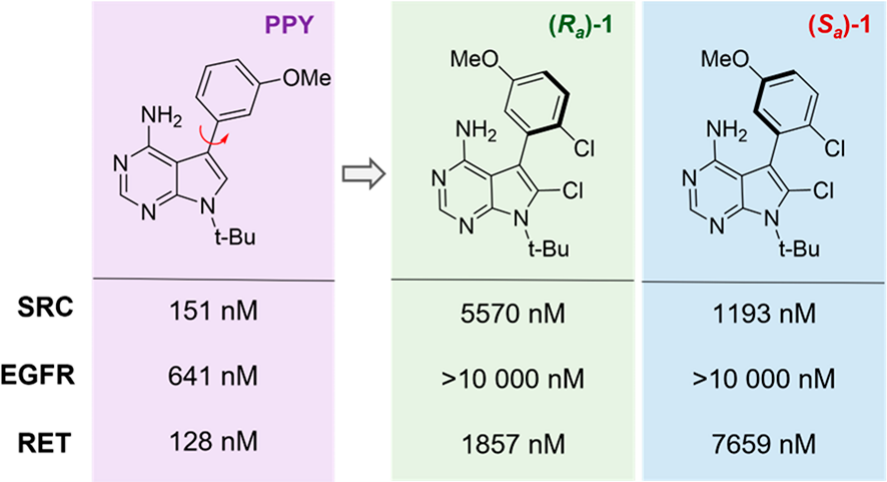
IC_50_s of atropisomerically stable analogs of PPY -based kinase-inhibiting scaffolds.

The prevalence of atropisomerism in modern drug discovery and the realization that many class-1 atropisomers bind their target in near-perpendicular conformations led our group to hypothesize that introducing class-3 atropisomerism into class-1 atropoisomeric scaffolds could lead to improvements in target selectivity by precluding off-target effects caused by the inhibition of proteins that preferred other conformations. We obtained a proof of principle in early work from our group where we designed class-3 atropisomers based on the privileged but promiscuous pyrrolopyrimidine (PPY) scaffold which is closely related to the venerable pyrazolopyrimidine (PP) class of kinase inhibitors.^1,38^ In this work, we observed that the class-3 atropisomeric analogs displayed improved kinase selectivity when compared to a class-1 interconverting “parent” molecule. Importantly, the atropisomers displayed different kinase inhibition profiles from one another, with the (*R*_a_) atropisomer inhibiting RET as its major target and the (*S*_a_) atropisomer inhibiting SRC and ABL (Figure 6). In essence, this work demonstrated that the promiscuous activities of a class-1 atropisomer could be decoupled to the different atropisomeric conformations.

**Figure 7:**
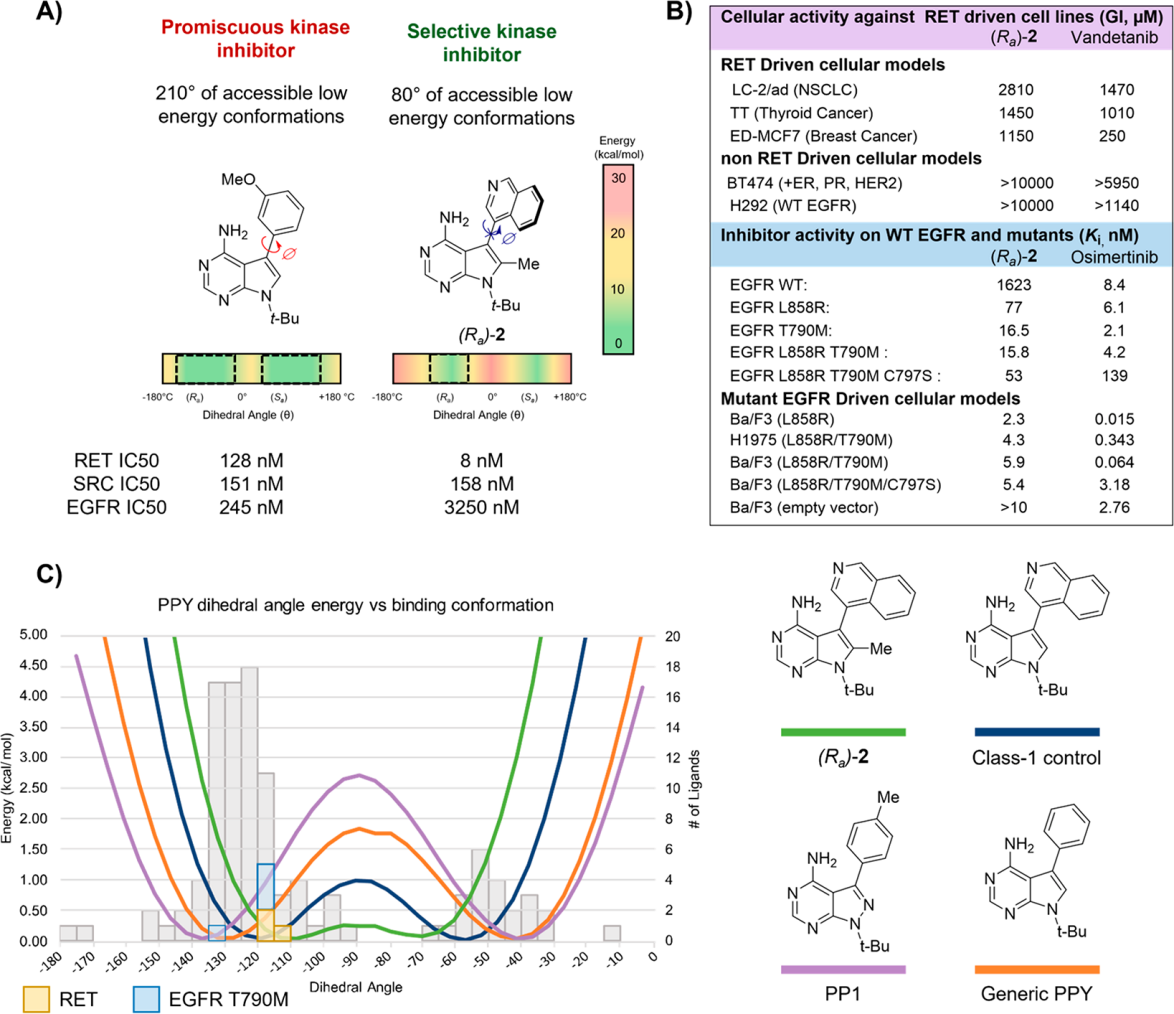
(A, B) Leveraging atropisomerism to obtain a selective inhibitor of RET. (C) A conformational map of PPY/PPs bound to different kinases sheds light on how introducing class-3 atropisomerism can improve target selectivity. The small-molecule conformations are measured from cocrystal structures available in the Protein Data Bank (PDB). The conformational energy profiles were calculated in the gas phase using density functional theory (B3LYP) with the 6-31G(d) basis set implemented.

Intrigued by the selectivity of the (*R*_a_) atropisomer toward RET kinase, an emerging therapeutic target for diverse cancers,^39−43^ we set out to optimize these compounds for RET, quickly arriving at compound (*R*_a_)-**2**^3^ (Figure 7A), which possesses low single-digit nM activity toward RET and orders-of-magnitude selectivity for RET over other kinases (e.g., VEGFR, EGFR) whose off-target inhibition is thought to be the source of adverse events in patients.^44^ This selectivity extended to cells in which (*R*_a_)-**2** possessed low μM activities against models of RET-driven cancers (Figure 7B). These activities were comparable, and in some cases improved, to those of promiscuous RET inhibitor vandetanib, the standard of care for RET driven cancers. Notably, vandetanib possessed activities toward RET independent cell lines, while (*R*_a_)-**2** did not, highlighting the improved selectivity of (*R*_a_)-**2**.

To understand the origin of this selectivity, we “mapped” the bound conformations of 109 PPY or similar PP ligands bound to kinases in cocrystal structures available in the protein database. As the majority of examples in this data set were pro-atropisomeric, we plotted the set across 180° to ensure a robust data set. Surprisingly, we observed that the bulk of conformational space about the axis was sampled by different kinases, which is demonstrated by the bar chart in the background of Figure 7C, where each bar represents the number of ligands bound in a given range of dihedral conformations.

Comparing the bound conformations with the predicted conformational energy profiles (CEPs) for different PP/PPYs offered evidence that the major driver of improved RET potency and selectivity for (*R*_a_)-**2** was the preorganization of the axis into a subset of conformations that were ideal for RET but not for other kinases. For example, the three RET structures in the data set revealed that the ligand (PP1 in each case) bound RET (2IVV, 5FM2, and 5FM3) with dihedral angles which are at or near the predicted local minimum for (*R*_a_)-**2** but correspond to destabilized conformations of PP1. The increased selectivity could then be explained by the narrower range of low-energy conformations available to (*R*_a_)-**2**. For example, of the 109 bound ligands in the analysis, 85 and 91% fell within the low-energy window (within 1.36 kcal/mol of local minima) of PP1 and a PPY with no ortho substitution, respectively. On the other hand, only 60% of the kinase-bound ligands fell within the low-energy conformations of (*R*_a_)-**2**, with ∼20% of the precluded ligands corresponding to those of the other (*S*_a_) atropisomer.

These observations led to the hypothesis that preorganizing the CEPs of promiscuous class-1 axes toward the preferred conformations of a target would allow for the “programming” of the scaffold’s selectivity toward that target. To obtain data in support of this, we analyzed our conformational map and found that EGFR mutants, but not WT-EGFR (wild-type EGFR), bound PP/PPYs in similar conformational ranges to RET. In essence, the conformational map predicted that (*R*_a_)-**2** would have secondary activities toward EGFR mutants but not the wild type. We found this prediction intriguing as acquired drug resistance to covalent inhibitors and side effects caused by the off-target inhibition of WT-EGFR have represented challenges in the mutant EGFR inhibitor field.^45,46^ In line with this prediction, we found that (*R*_a_)-**2** had little WT-EGFR activity but possessed low nanomolar activities toward oncogenic EGFR mutants (Figure 7B). (*R*_a_)-**2**’s mutant selectivity over WT-EGFR compares favorably to that of osimertinib, the standard of care for mutant EGFR cancers, particularly for the L858R/T790M/C797S mutant which has proven to be a challenge to the drug.^47^

These studies suggest that dihedral conformations about a potential atropisomeric axis play a key role in the recognition of small molecules by proteins and that preorganizing a promiscuous small molecule into the preferred conformations of a target can reprogram the scaffold’s selectivity toward that target. While similar conformational effects have been previously discovered serendipitously and are often referred to as the “magic methyl effect”,^48^ this work provides a predictive approach that can empower the application of these conformational effects toward selectivity optimization.

**Figure 8:**
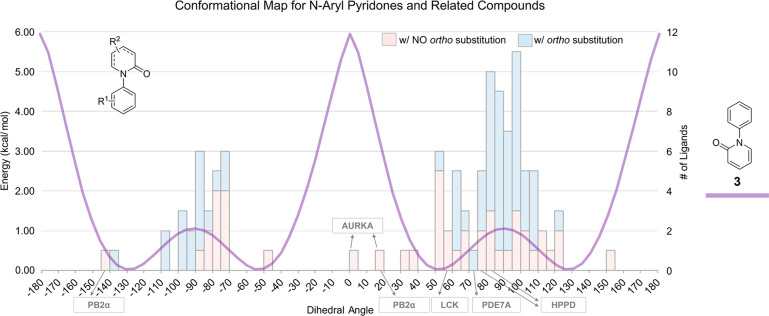
Conformational map for *N-*aryl pyridones and related compounds. The small-molecule conformations are measured from protein/small-molecule cocrystal structures available in the Protein Data Bank (PDB). A table including each example is included in the Supporting Information. The conformational energy profiles were calculated in the gas phase using density functional theory (B3LYP) with the 6-31G(d) basis set implemented.

As class-1 atropisomerism is ubiquitous in drug discovery, there are myriad promiscuous scaffolds whose selectivity could be improved toward a given target via conformational control about a potential atropisomeric axis. As such, we have generated conformational maps for other privileged potentially atropisomeric scaffolds.^49^ For example, we have generated conformational maps for potentially atropisomeric *N-*aryl pyridones and related scaffolds, of which the FDA-approved drug sotorasib is a member. We found 110 unique cocrystal structures of these chemotypes bound to different targets. Measuring the dihedral angles and plotting the ligands’ binding conformations overlaid with their CEP (Figure 8) revealed a similar conformational landscape to the PP/PPY scaffolds albeit with a few notable differences. For example, the shorter bond length of the C–N axis coupled with the geometries that result due to both rings about the axis being six-membered^50^ resulted in the low-energy conformational ranges about the axes being shifted toward more orthogonal conformations compared to PP/PPYs. Furthermore, differential ortho substituents were more common in this data set, allowing us to use a full 360° plot to separate entries by atropisomeric conformation, with 0 to 180° representing one set of atropisomeric conformations and 0 to −180° representing the enantiomeric conformations. This data set suggests that many of these scaffolds would benefit by being rigidified into a stereochemically defined class-3 atropisomer.

**Figure 9:**
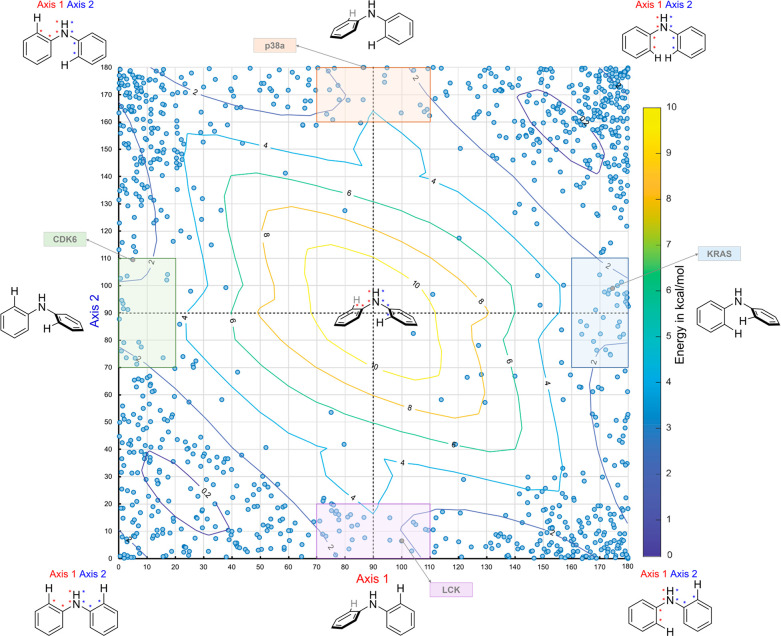
Conformational map for diarylamines. Potentially atropoisomeric conformations are highlighted in different colors, with some exemplary targets listed for each chiral conformation. The small-molecule conformations are measured from protein/small-molecule cocrystal structures available in the Protein Data Bank (PDB). A table including each example is included in the Supporting Information. The conformational energy profiles were calculated in the gas phase using density functional theory (B3LYP) with the 6-31G(d) basis set implemented.

We have also constructed a conformational map for diarylamines, which are among the most privileged scaffolds in modern drug discovery and also possess two contiguous potentially atropisomeric C–N axes. A search of the PDB revealed over 1600 unique small-molecule/protein cocrystal structures, with myriad examples bound to their target in potentially atropisomeric conformations. We generated a conformational map for diarylamines by sorting each ligand by its dihedral conformation about both axes and overlaying 3D energy coordinates of simple diarylamine scaffolds (Figure 9). This conformational map reveals that diarylamines sample diverse conformational space while binding to their diverse targets, with lower-energy conformations where both axes are in nearly planar conformations being the most abundant. Despite this, of the 1600+ entries, we found that more than 100 ligands, including FDA-approved drugs Bosutinib, Imatinib, and mefenamic acid, had diarylamines that bound their targets in higher-energy conformations in which one of the axes was planar and the other axis was in a nearly orthogonal atropisomeric conformation. These conformations are of particular interest as work from Kawabata^19^ and our laboratory (*vide infra*)^4,19^ suggests that it is possible to obtain stable diarylamine atropisomers in these conformations.

Despite the abundance of diarylamines in modern chemistry, examples of stable diarylamine atropisomers have remained rare as the contiguous nature of the C–N axes allows for lower-energy concerted gearing mechanisms of racemization in which the simultaneous rotation of both axes allows access to low-energy pathways of racemization. Kawabata was the first to disclose atropisomerically stable diarylamines when his group discovered that diarylamines that possess an intramolecular hydrogen bond between an *ortho*-imine and the diarylamine N–H^51,52^ existing as “near” class-3 atropisomers. It is postulated that the intramolecular hydrogen bond prevented the lower-energy concerted gearing racemization pathway by locking one of the axes into a planar conformation. More recently, Clayden^18^ published a seminal study that explored the steric factors of the four ortho positions of acyclic diarylamines needed to obtain atropisomerically stable acyclic diarylamines without intramolecular hydrogen bonding, obtaining one compound with a Δ*G*^⧧^_rac_ value of 31.1 kcal/mol. Intrigued by the prospective atropisomers in our diarylamine conformational map and the aforementioned precedence of stereochemically stable diarylamines, we sought to determine if we could obtain class-3 atropisomeric analogs of pharmaceutically relevant diarylamines.^19^

**Figure 10:**
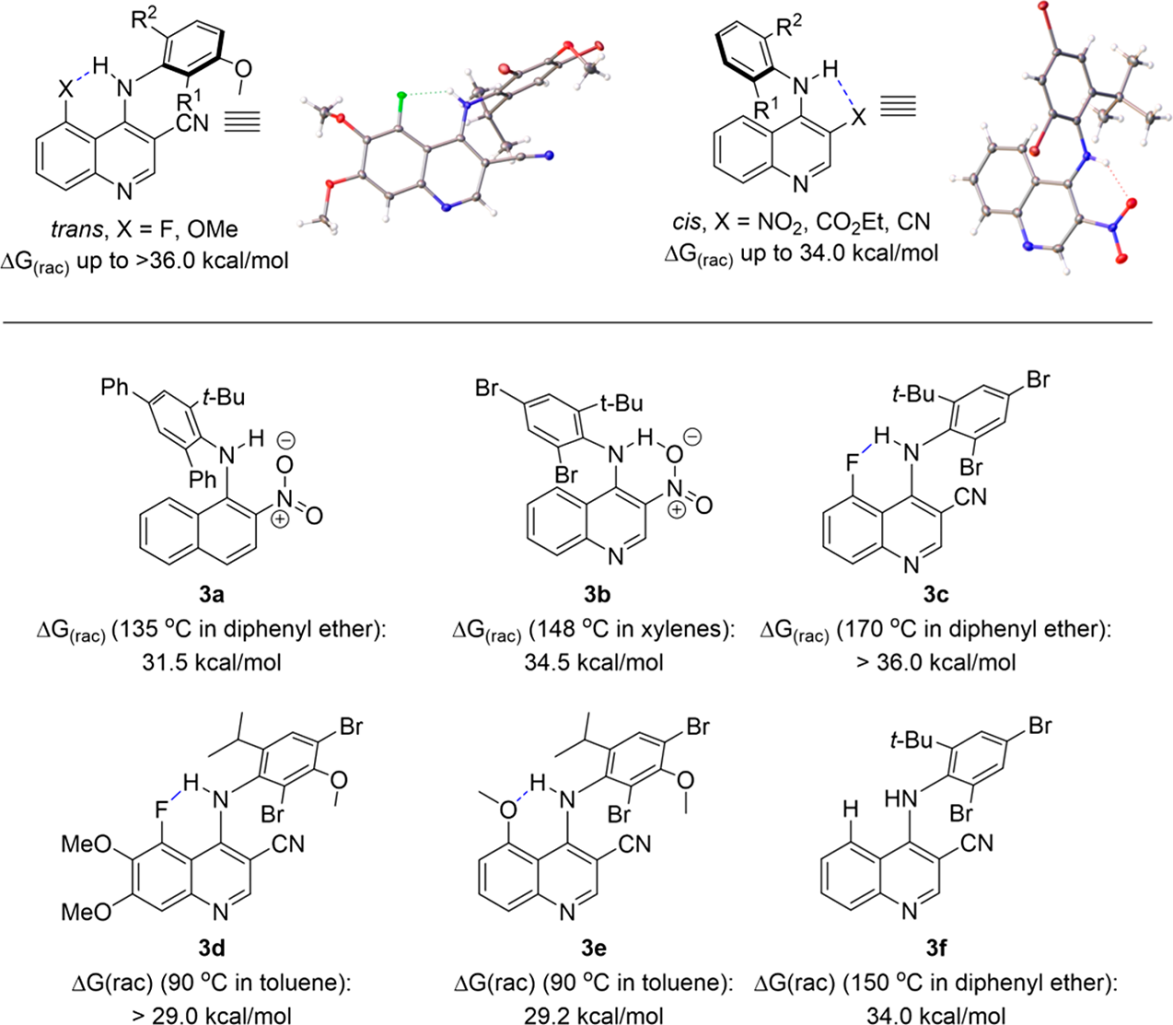
Atropisomerically stable diarylamines based on quinolines.

We initially evaluated *ortho*-nitro-containing quinoline **3a** (Figure 10) and observed a barrier to rotation of 31.5 kcal/mol, which is largely in line with Clayden’s system. When we evaluated analog **3b**, now based on a quinoline scaffold, we observed a drastic increase in stereochemical stability to 34.5 kcal/mol, with crystal structures offering evidence of an intramolecular hydrogen bond between the NO_2_ group and the diarylamine N–H in the ground-state conformations. We next evaluated analogs based on the core scaffold of the FDA-approved drugs Bosutinib and Neratinib^53^ that possessed a peri substituent that could lock the quinoline C–N axis into a single planar conformation via intramolecular hydrogen bonding and thus preclude the concerted gearing mechanism of diarylamine racemization. Inspired by work from Lectka^54^ on the hydrogen-bonding ability of fluorine, we initially evaluated *peri*-fluorine-substituted **3c** and observed no racemization after prolonged heating at 170 °C, suggesting that the barrier to rotation was greater than 36 kcal/mol. Intrigued by the high stereochemical stability of **3c**, we next studied analogs with smaller substitutions (i.e., **3d**) and different peri hydrogen-bonding acceptors (i.e., **3e**) and observed that they remained class-3 atropisomers with Δ*G*^⧧^_rac_ greater than 29 kcal/mol at 90 °C in toluene, a benchmark stability that is often considered to be stable enough for drug development. Control experiments suggested that intramolecular hydrogen bonding between the N–H and peri substituent contributed 2 to 3 kcal/mol to the barrier to racemization; however, the major driver of the unexpectedly high observed stereochemical stabilities was increased conjugation of the diarylamine lone pairs into the electron-poor quinoline, both stabilizing the planar conformations and shortening the axis.

## 4 Atropisomer Selective Synthesis of Pharmaceutically Relevant Scaffolds

The above approach toward selectivity often results in the need for enantiopure samples of atropisomers. While traditional resolution methods can furnish enantiopure samples from racemic mixtures, they can often be time-consuming, resource-intensive, and not practical in the context of structure optimization. These challenges have been given a recent spotlight as more class-3 atropisomers make it to the clinic, often requiring heroic efforts to meet the challenges of material throughput.^13,55^ While atroposelective methods have been studied for decades, they have largely focused on biaryls, leaving relatively few methodologies^22,56−58^ that are applicable to the other pharmaceutically relevant scaffolds. This has led our group to embark on a series of projects that focus on the development of atropisomer-selective methodologies toward pharmaceutically relevant scaffolds.

**Figure 11:**
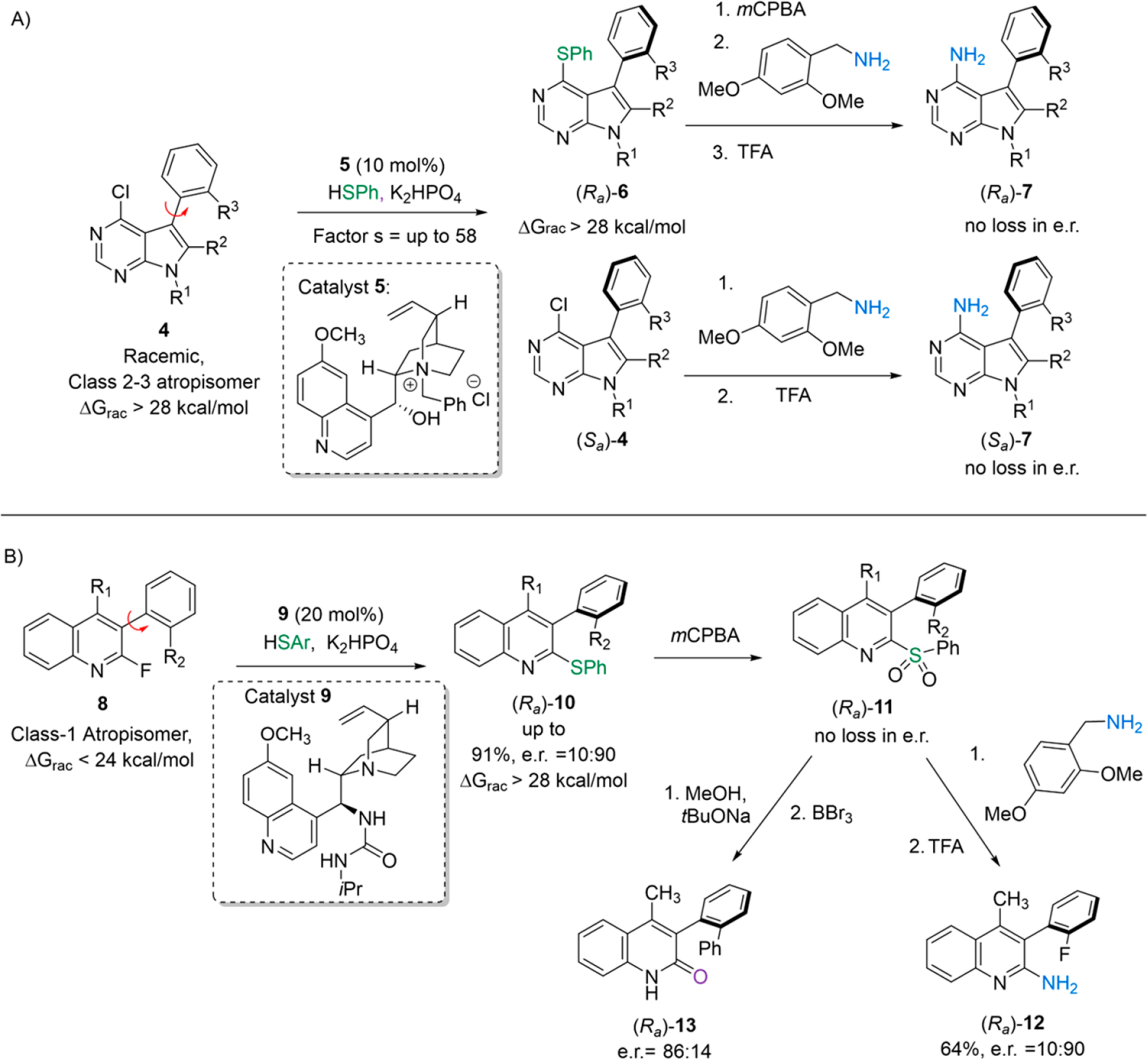
Atroposelective S_N_Ar toward pharmaceutically relevant scaffolds.

Inspired by several analyses on the most represented scaffolds and reactions in the pharmaceutical patent literature,^14,59^ we set out to develop atroposelective methodologies that employed S_N_Ar and related reactions on common aromatic and heteroaromatic scaffolds. In 2014, Smith and co-workers^60^ published a seminal atroposelective desymmetrization wherein ammonium salts derived from cinchona alkaloids were used to direct the S_N_Ar addition of thiophenols into pro-atropisomeric pyrimidines. We were intrigued by this chemistry as it had the potential to be applied to diverse heterocyclic frameworks and presented opportunities for further elaboration of the enantioenriched products directly into privileged scaffolds by leveraging the rich chemistry of sulfur.^61,62^ In 2018, we disclosed a kinetic resolution approach toward atropoisomeric PPY-based kinase inhibitors that proceeded through a chiral cation-directed S_N_Ar of thiophenols into PPYs^63^ (Figure 11A). Our optimal catalyst (**5**) and conditions worked well on diverse PPYs, often allowing for access to the products (**6**) and recovered starting materials (**4)** in greater than 95:5 e.r. at ∼50% conversion. We also developed processes to transform both the product and recovered starting material to the final kinase-inhibiting scaffold (**7**) with no racemization in a stereodivergent manner. This work allowed us to discover a new selective inhibitor of breast tumor kinase (BRK).

The above chemistry proceeded as a kinetic resolution because the substrate and product had similar stereochemical stabilities (∼28 kcal/mol). We hypothesized that substrates with a smaller leaving group ortho to the axis could be amenable to dynamic kinetic resolution (DKR) as the axis could racemize during the course of the reaction until a larger nucleophile displaced it. Indeed, we found that many 3-aryl-2-fluoroquinolines (**8**) were amenable to atroposelective DKR when thiophenols were utilized as the nucleophile (Figure 11B).^64^ Our optimal catalyst (**9**) and conditions yielded products (**10**) in up to 91% yield and 91:9 e.r. (>97:3 e.r. after trituration). When substrates had larger substituents adjacent to the axis, we observed classical KRs with *s* factors of up to 27. Importantly, we were able to transform the products to 2-aminoquinolines (**12**) and 2-quinolones (**13**) with minimal observed racemization. Taken together, our work in this area demonstrates that atroposelective S_N_Ar represents a flexible approach to accessing atropisomerically enriched, pharmaceutically relevant scaffolds.

One of the limitations of S_N_Ar in the context of atroposelective DKR is the need for a leaving group adjacent to the axis that in many instances results in the substrate not having a sufficiently low barrier to rotation to allow for the needed level of racemization during the course of the reaction. Indeed, the aforementioned atroposelective syntheses of PPYs proceeded as kinetic resolutions, and 3-arylquinoline substrates with larger ortho substitutions displayed significant kinetic resolution character as well. This has led us to simultaneously explore atroposelective vicarious nucleophilic substitutions (VNS) and related reactions. In VNS, a small hydrogen atom is replaced by a larger nucleophile, which would allow for a wider scope of substrates and scaffolds that are capable of undergoing atroposelective DKR.

**Figure 12:**
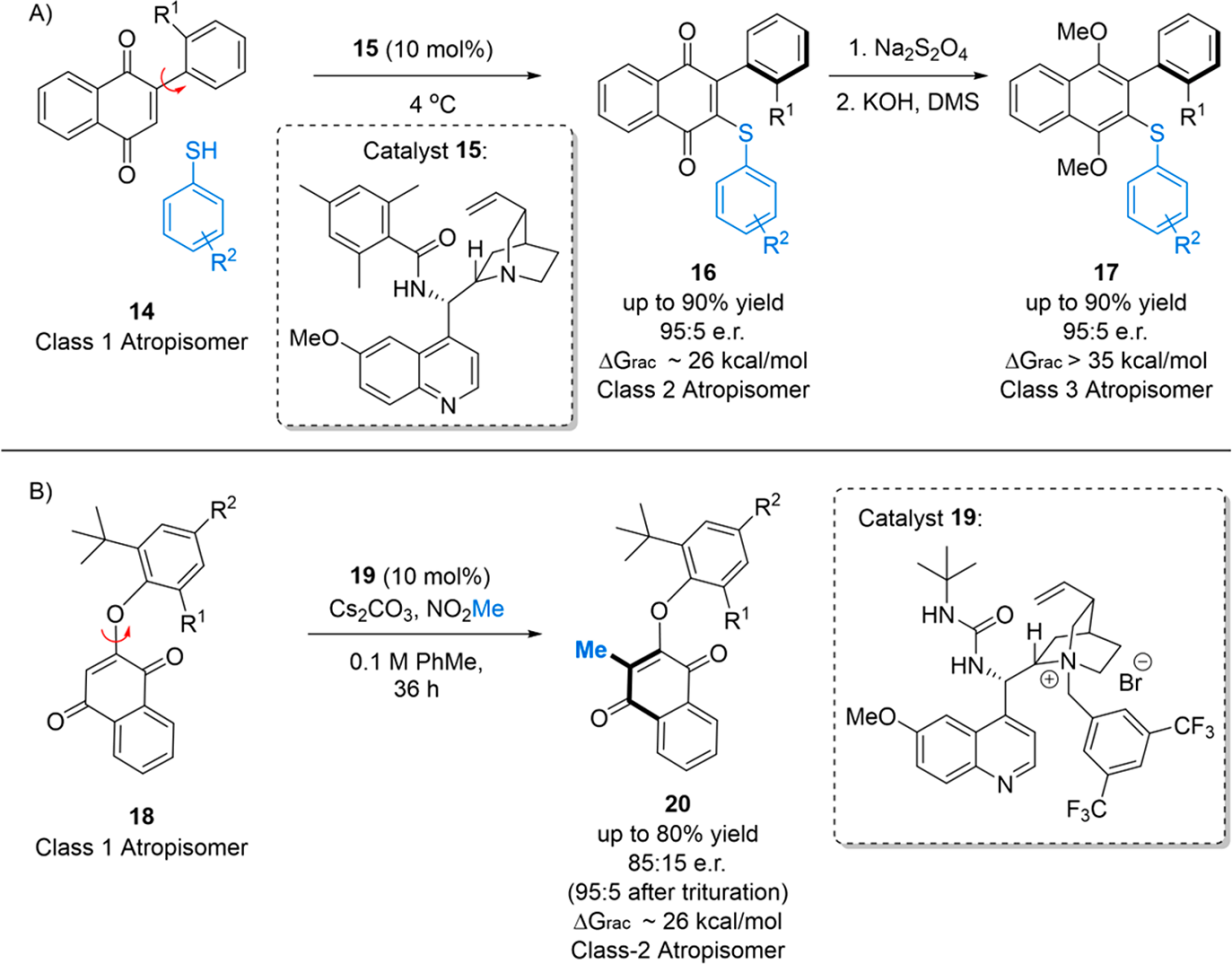
Atroposelective VNS toward biaryls and *O*-arylquinoids.

In seminal work by Tan,^65^ it was discovered that electron-rich aromatics could be added to quinones to give atropisomerically enriched biaryls via a net-VNS process. Inspired by this, we postulated that aryl-substituted naphthoquinones that exist as class-1 atropisomers such as **14** (Figure 12A) could be substrates for atroposelective DKRs where a nucleophile adds adjacent to the aryl group to transform the axis to a class-3 atropisomer. In support of this, we observed that quinine-derived catalysts possessing a sterically hindered benzamide off of the C-9 position (**15**) could affect the addition of diverse thiophenols into aryl-substituted naphthoquinones to give substituted quinone products (**16**) in good yields and selectivity; however, they existed as class-2 atropisomers. Subjecting the products to a reductive workup allowed us to isolate biaryls **17** that existed as class-3 atropisomers (∼36 kcal/mol). The dramatic increase in the stereochemical stability of hydroquinones compared to that of quinones is in line with previous observations in the context of natural products.^66^ In the end, our optimal conditions followed by a reductive alkylative workup allowed us to obtain stereochemically stable products in >90% yields and enantioselectivities above 95:5 e.r.

We have also studied a similar VNS approach toward *O*-arylquinoids, a scaffold that is related to diaryl ethers. Despite atropisomeric diaryl ethers being observed in natural products such as vancomycin and class-1 atropisomeric diaryl ethers being common chemotypes in drugs (i.e., regorafenib), the asymmetric syntheses of diaryl ethers and related compounds have been understudied, likely because, as with diarylamines, diaryl ethers possess two contiguous axes that allow for a low-energy concerted gearing racemization pathway. In seminal work, Clayden has found that diaryl ethers with four ortho substituents and at least one tertiary alkyl group (i.e., *t-*Bu) can exist as stereochemically stable class-3 atropisomers.^67^ Clayden and collaborators subsequently leveraged these findings to develop a biocatalytic desymmetrization that allowed access to enantioenriched diaryl ethers.^68^

Inspired by these precedents, we designed a class of *O*-aryl quinoids that could exist as isolable class-2 atropisomers (Figure 12B) and evaluated different VNS-like strategies toward an atroposelective synthesis of the scaffold. Inspired by work from Mukherjee,^69^ we found that we could affect atroposelective alkylations on substrates such as **18** using nitroalkane as the alkyl source to give class-2 atropisomeric products such as **20**.^70^ The optimal catalysts proved to be sterically hindered ureas containing quinine derivatives (i.e., **19**) and could affect the alkylation in good yields and moderate to good enantioselectivity (up to 85:15 e.r. and 95:5 e.r. after trituration). The moderate enantioselectivity could perhaps be explained by the lower stereochemical stabilities (barrier to rotations of 25–28 kcal/mol) of the products that could allow for some racemization over the course of the reaction.

**Figure 13:**
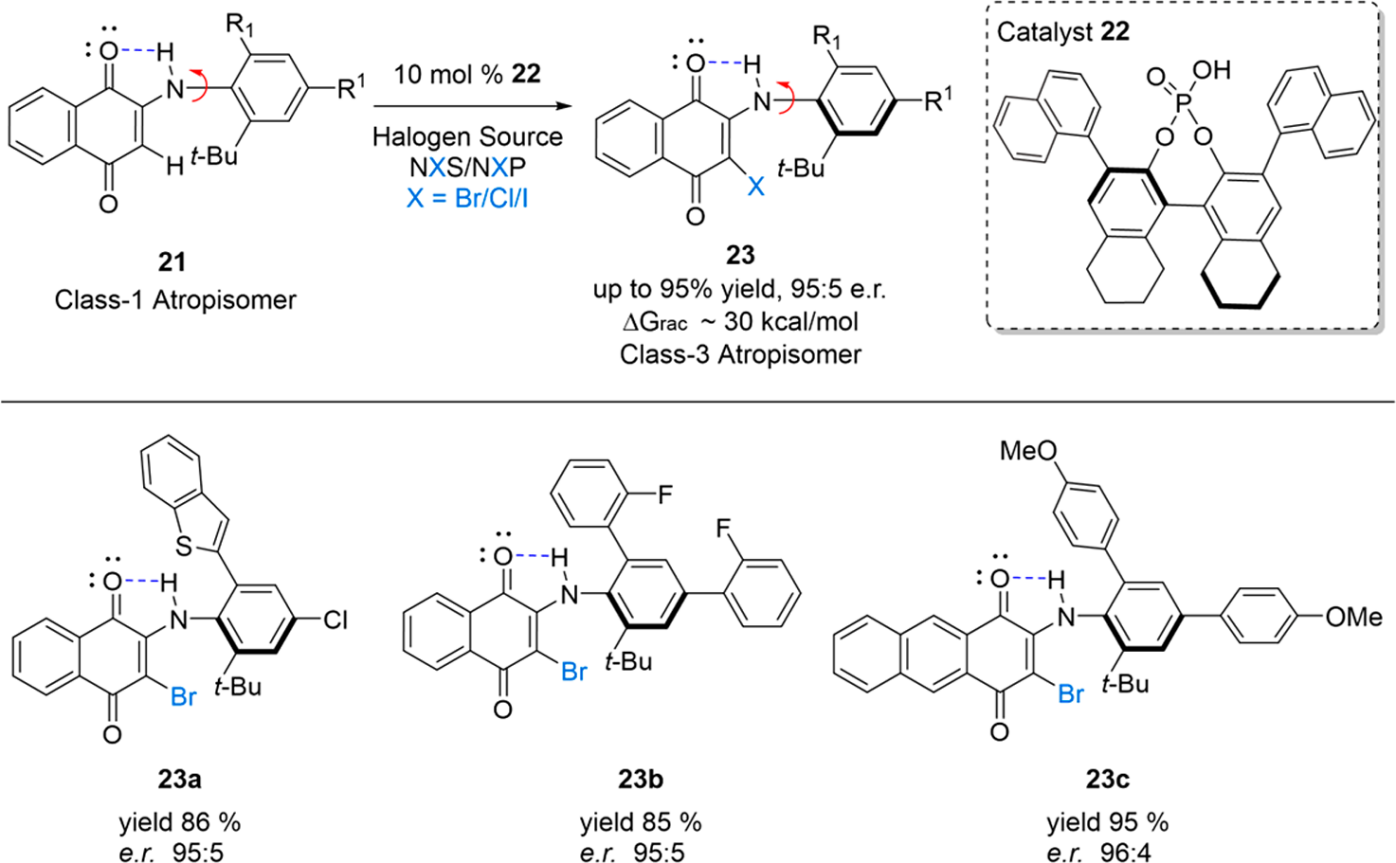
Atroposelective synthesis of *N-*aryl quinoids.

The ability to obtain class-2 atropisomeric *O*-aryl quinoids in an enantioenriched manner led us to explore *N-*aryl quinoids which are a related scaffold to diarylamines, a common scaffold in drug discovery that, as discussed in previous sections, represents a long-running interest of our group. While there has been recent interest in the development of asymmetric methodologies toward atropisomers based on C–N axes, the majority of the effort has focused on anilides and related cyclic scaffolds.^71−74^ The lack of precedence for the asymmetric syntheses of diarylamines and related scaffolds is likely due to the two contiguous C–N axes leading to a complex conformational profile (as shown in Figure 9) that also allows for a lower-energy concerted gearing mechanism of racemization. Inspired by our work with *O*-aryl quinoids as well as work from Kawabata on leveraging intramolecular hydrogen bonding to obtain stereochemically stable diarylamines, we designed a series of *N-*aryl quinoids (Figure 13) that existed as low class-3 atropisomers. Next, inspired by work from Miller^20^ and Akiyama,^21^ we developed a chiral phosphoric acid-catalyzed bromination to transform class-1 atropisomeric substrate **21** into class-3 atropisomeric product **23**. Our optimal catalyst **22** was able to effect this bromination in good yields and enantioselectivity (90% yield, e.r. > 95:5) across a large scope of *N-*aryl quinoids, such as **23a**, **23b**, and **23c**. This work represented the first example of an asymmetric synthesis of any scaffold related to acyclic diarylamines, and many of the lessons learned during this work allowed us to design the stereochemically stable diarylamines described in Figure 10. It is also likely that the atroposelective bromination strategy is applicable to direct diarylamine scaffolds.

## 5 Conclusions and Outlooks

Atropisomerism is a dynamic type of chirality that is becoming increasingly ubiquitous in modern drug discovery and other fields. Ours and others’ work over the past decade has demonstrated that atropisomerism can often be leveraged to improve various properties of a small-molecule pharmaceutical lead, with our group focusing primarily on improving the target selectivity of kinase inhibitors and other promiscuous classes of small molecules. Subsequent work where we “mined” the Protein Data Bank for prospective atropisomers bound to different protein targets led to the realization that the sampled dihedral conformations about a prospective atropisomeric axis played a key role in target recognition and that preorganizing a potentially atropisomeric axis into a desired target’s preferred conformational window can reprogram the scaffolds’ selectivity toward that target. This finding not only explains how introducing stable atropisomerism can improve target selectivity but also informs us of opportunities wherein controlling the conformational profile about a prospective atropisomeric axis can lead to improvements in potency and selectivity across diverse privileged pharmaceutical scaffolds. While similar conformational effects have been previously discovered serendipitously and are often called “magic methyls”,^48^ this work can perhaps provide a predictive data-based approach that can empower selectivity optimization and represent a new tool for medicinal chemists.

As atropisomerism becomes more prevalent in drug discovery, there is an increasing need for methodologies to obtain enantioenriched samples of pharmaceutically relevant atropisomers. This has led us to undertake several projects that strive to develop atroposelective methodologies that leverage the most commonly employed reactions in modern drug discovery, with an emphasis on atroposelective methodologies that can directly lead to privileged biologically active scaffolds. Our work on atroposelective S_N_Ar in particular has allowed us to access many pharmaceutically relevant scaffolds in an enantioenriched fashion (i.e., PPYs, quinolones, and aminoquinolines). This has also led us to explore the potential for introducing class-3 atropisomerism into pharmaceutically privileged scaffolds that are not traditionally thought of as atropisomeric, such as diarylamines. Beyond atroposelective catalysis, there are also opportunities to leverage the dynamic nature of atropisomerism to allow for efficient access to atropisomerically pure compounds at scale, as recently demonstrated in work by Mirati Therapeutics^55^ wherein they leveraged a traditional diastereomeric resolution with in-line flash racemization of the undesired atropisomer to achieve a DKR in the synthesis of MRTX-1719. Moving forward, we hope that the field of atroposelective catalysis will turn to the pharmaceutical industry when looking for inspiration of what scaffolds toward which to develop atroposelective methodologies and embrace the challenge of developing chemistry that will have direct applications to the pharmaceutical realm.

## Supporting Information Available

Poster of FDA-approved atropisomeric pharmaceuticals; conformational data obtained from PDB analyses (PDF)
