# Supplementary material for: Atropisomerism in the Pharmaceutically Relevant Realm
Source: Acc Chem Res. 2022 Sep 26;55(20):2904–19. doi: 10.1021/acs.accounts.2c00500 (PMC9583608; doi:10.1021/acs.accounts.2c00500)
Supplement: Supplementary file 1 — ar2c00500_si_001.pdf [file ar2c00500_si_001.pdf]

Supporting Information

Atropisomerism in the Pharmaceutically Relevant Realm

Mariami Basilaia*, Matthew H. Chen*, Jim Secka*, Jeffrey L. Gustafson*

*Department of Chemistry and Biochemistry, San Diego State University, 5500 Campanile Dr., San Diego, California 92182-1030, USA

Email: jgustafson@sdsu.edu

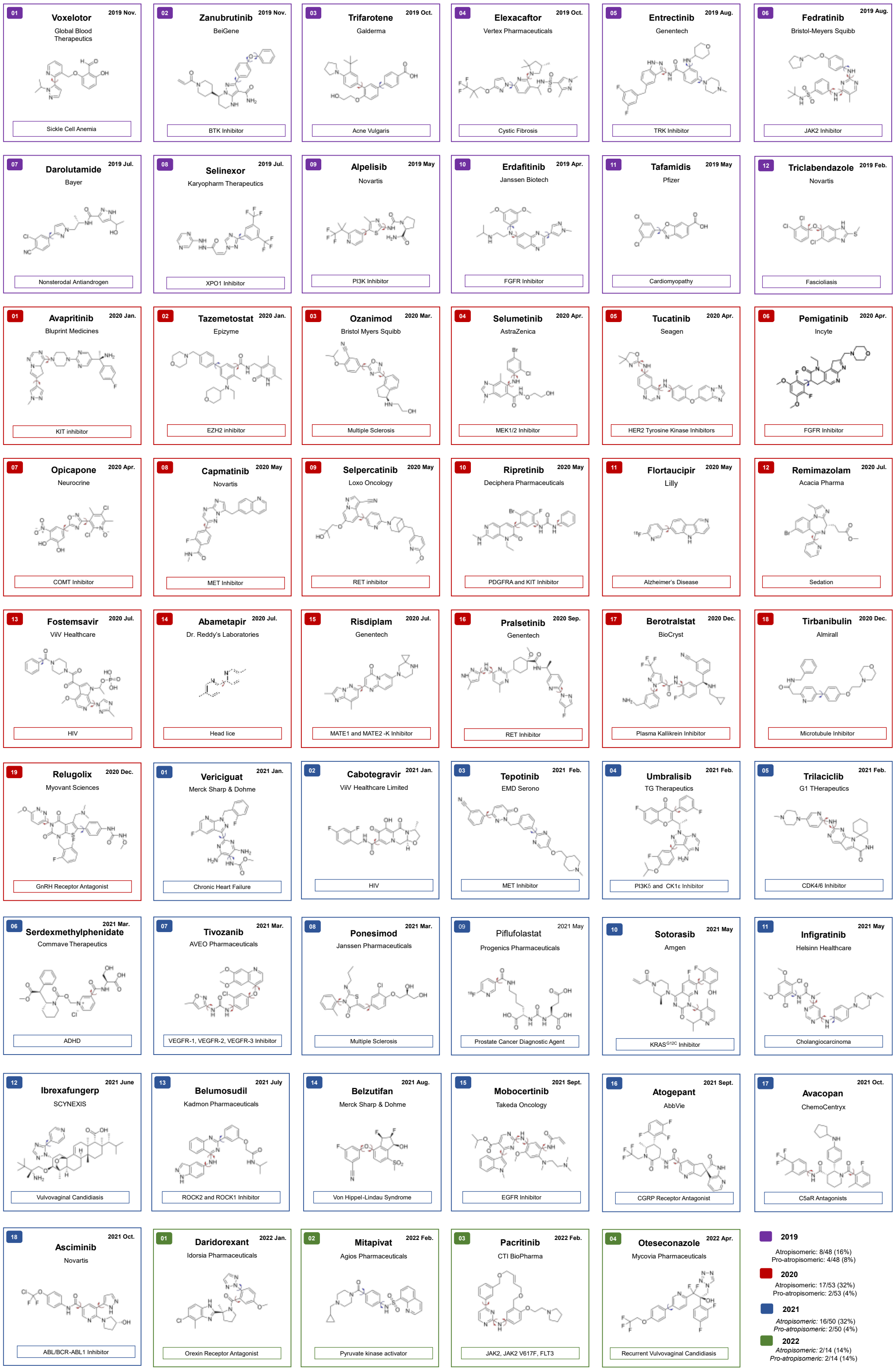

Figure S1. Examples of FDA-approved drugs as of June 2022 that possess a prospective atropisomeric axis. Atropisomeric axes are denoted by red arrows. Pro-atropisomeric axes are denoted by blue arrows.

Table S1. Examples of Protein Data Bank ligand conformation analysis for pyridones.

Chemical ID	PDB ID	Protein	Torsion Angle
7MP	2HIW	Abl	-88.419998
IR2	6GIN	ACVE1	153.60001
A5Y	6DUM	ALDH1A1	95.919998
M39	5TEI	ALDH1A1	124.02
P9J	3P9J	AURKA	18.59
P9J	3W16	AURKA	36.689999
6MV	5JRS	BTK	-70.07
7G7	5P9H	BTK	80.559998
2V2	4OTQ	BTK	86.660004
6XL	5KUP	BTK	86.660004
7G6	5P9G	BTK	88.18
2V3	4OTR	BTK	89.639999
GMW	6HRT	BTK	93.629997
3OV	4RFZ	BTK	93.910004
73T	5T18	BTK	96.389999
GMQ	6HRP	BTK	97.080002
KLM	6NFH	BTK	100.8
38M	3F9N	Chk1	61.860001
9XK	5OKT	CSNK1D	108.03
FQC	5EAF	CYP51	82.900002
LM7	6O5J	DAD2	80.230003
B6N	6EIS	DYRK1A	-87.690002
3LH	4WD5	EGFR T790M	52.950001
F62	5GMP	EGFR T790M	105.79
WQQ	5ABW	ELA2	100.62
C07	4G2F	EphA3	-108.58
H21	2VVV	FXa	-88.339996
LZH	2VWL	FXa	-84.339996
455	2XBW	FXa	-80.379997
IVK	2XC4	FXa	-79.519997
RR8	2XBX	FXa	-78.309998
OYJ	2XC5	FXa	-77.339996
H22	2VVU	FXa	-76.040001
LZG	2VWO	FXa	-74.029999
XBV	2XBV	FXa	-73.779999
H25	2VWN	FXa	-73.730003
8NC	2XC0	FXa	-72.769997
LZF	2VVC	FXa	89.650002
FXA	3CEN	FXa	94.150002
230	2PHB	FXa	97.260002

L1D	2W3K	FXa	98.120003
ME1	2P93	FXa	98.260002
LZI	2VWM	FXa	100.38
ME5	2P95	FXa	107.92
ME4	2P94	FXa	112.06
L1C	2W3I	FXa	123.55
6ZQ	5L1E	GluA2	77.580002
6ZP	5L1F	GluA2	121.79
HR1	2Q6C	HMGCR	96.309998
92X	5YWK	HPPD	-89.519997
94L	5YY7	HPPD	96.860001
94L	5YY6	HPPD	105.28
9G4	4D0S	InhA	54.360001
41I	4D0R	InhA	77.879997
MOV	6OIM	KRAS	-85.510002
OHY	6PGP	KRAS	-84.739998
AM9	3BYO	Lck	78.949997
353	6SDD	Met	64
1FN	3CE3	Met	65.080002
353	3F82	Met	66.139999
L1X	4EEV	Met	72.370003
17G	4JSX	mTOR	81.269997
ORH	3KWB	p38a	-143.95
AA0	4AA0	p38a	-99.150002
QC0	4AA4	p38a	-96.940002
AAV	4AAC	p38a	-96.25
NQB	4AA5	p38a	-91.839996
222	2I0H	p38a	-77.550003
F4C	3K3J	p38a	54.82
0OK	4EH2	p38a	82.839996
I45	3HLL	p38a	100.97
29A	3ROC	p38a	108.11
IY1	6O9E	p66rt	63.139999
3QI	4ANP	PAH	-90.779999
6BZ	5IME	PAK1	65.650002
QLN	6Q9N	PBP2a	-135.28999
QNZ	4CJN	PBP2a	32.43
PVM	6U36	PCSK9	119.1
4PX	5C2H	PDE10A	79.529999
4PX	5DH4	PDE10A	80.349998
4PX	4ZO5	PDE10A	80.440002
4PX	5C2E	PDE10A	81.790001
4Q0	4ZO5	PDE10A	97.839996

D71	3G4I	PDE3D	60.970001
D71	3G4G	PDE3D	61.939999
TC8	3G3N	PDE7A	92.790001
NRF	5MOG	PDS1	-45.040001
ZZQ	2WXQ	PI3Kd	-107.91
68R	5I6U	PI3Kd	79.93
S30	2WXI	PI3Kd	80.639999
40L	4XE0	PI3Kd	82.139999
IC8	2X38	PI3Kd	85.800003
ZZN	2WXG	PI3Kd	87.809998
ZZO	2WXH	PI3Kd	89.669998
039	2WXF	PI3Kd	90.910004
EO5	6G6W	PI3Kd	94.029999
67T	5I4U	PI3Kd	97.129997
039	2CHW	PI3Kg	88.300003
76C	5T7F	PIK3d	-86.559998
77C	5T8I	PIK3d	93.279999
7KA	5M6U	PIK3d	98.120003
1FN	6OKO	RIPK3	50.720001
PFA	1N46	THRB	110.49
5U4	5F20	Tyk2	-71.639999
KZJ	6NSL	Tyk2	104.22
JW7	4BTW	VAP1	1.5700001
WF8	4BTX	VAP1	52.290001
JWF	4BTY	VAP1	55.360001
C52	3CPC	Vegfr	53.200001
C19	3CP9	Vegfr	59.029999

Table S2. Examples of Protein Data Bank ligand conformation analysis for diarylamines.

Chemical ID	PDB ID	Protein	Torsion Angle_1	Torsion Angle_2
03P	3RCD	Erbb2	1.314374	55.89254
03P	3POZ	EGFR	6.769298	48.96539
03P	3W2O	EGFR	15.32398	43.55212
03Q	3PP0	Erbb2	5.192722	52.7892
048	3UVP	p38a	5.192722	179.1281
04K	3PJ2	BTK	11.36044	150.9587
04T	4H80	Aldh3A1	26.26856	70.96517
066	3GWT	Pde4B	60.67017	10.29571
06F	3UVR	p38a	61.2398	178.2488
071	3KB7	Plk1	175.148	146.6291
07J	3TT0	Fgfr1	19.1916	116.3234
0F0	3V6S	JNK3	165.9578	165.4695
0F4	3V5Q	Ntrk3	150.8097	170.3127
0HV	4DBS	Akr1C3	20.47499	160.4943
0JF	4DCE	Alk	146.7586	0.245104
0KO	4DK5	Pik3g	135.4525	171.5736
0N5	4E1K	Glmu	3.209271	129.2718
0SB	4F4P	Syk	173.8817	175.0663
0TO	4FEX	Abaye3578	164.9238	166.4642
0UN	5X2K	EGFR	151.3456	9.605191
0UN	3IKA	EGFR	153.1344	178.5192
0VE	4FYN	Syk	175.5475	178.7115
0VF	4FYO	Syk	170.2166	178.7905
0VG	4FZ6	Syk	0.218583	174.3331
0VH	4FZ7	Syk	14.90951	163.5638
0VU	4FUL	Pik3g	160.6564	22.08346
0WM	4G5J	EGFR	6.568463	176.4853
0WN	4G5P	EGFR	18.61861	2.660723
0WN	4G5J	EGFR	29.41213	17.38979
0X3	4ZQD	Arnt	19.72552	69.67466
0X3	4GHI	Epas1	53.82567	11.58543
0XB	4GS9	Epas1	70.32598	179.8657
0XF	4GFG	Syk	3.203089	0.503903
0XG	4GRB	CK2	166.3067	177.731
10Z	4H58	Braf	5.266072	0.459141
11V	4O2P	Src	156.3976	83.83662
13K	3TJP	Pik3g	15.33679	177.725
14K	4FLH	Pik3g	127.6239	176.9991
16K	4EK8	Cdk2	1.15903	4.435254

16X	2X4F	Mylk4	20.60114	135.3741
17C	4IJ1	Trpd	6.572619	31.48994
17C	3QQS	Trpd	177.836	35.50621
185	1SUQ	HIVrt	166.301	0.364774
18K	3SW4	Cdk2	175.8591	168.3703
19K	3SW7	Cdk2	173.8789	169.2326
19T	4MYQ	Pde4B	8.17699	144.8935
1BM	2HK5	Hck	73.96303	178.7978
1BU	3F3W	Src	149.9383	167.9211
1BU	3GCQ	p38a	157.1279	100.2428
1BU	3F3V	Src	163.5418	82.15852
1FV	6QAU	Ulk2	162.776	160.4787
1FV	4IWQ	Nak	174.2829	176.7899
1FV	4IM0	Nak	179.6956	2.85141
1H4	4IWO	Tbk1	156.2329	0.662102
1J3	4J52	Plk1	172.3601	0.826271
1J4	4J53	Plk1	172.7977	179.2451
1N6	4JX7	Pim1	134.7745	9.148812
1N9	2OFU	Lck	1.870018	152.1166
1RE	4KKO	HIVrt	5.201545	8.322719
1S8	4KNR	Glmu	11.06603	37.07216
1S9	4KNX	Glmu	3.713797	38.96923
1WY	4LI5	EGFR	179.6529	157.6688
20K	4FKO	Cdk2	172.9363	168.0299
23D	2CDZ	PAK4	4.725226	19.57754
23D	2F57	PAK7	23.11107	4.366521
23D	6GUF	Cdk2	136.0636	7.978904
24A	2BDF	Src	5.325873	163.9019
24V	4TTH	Cdk6	142.6234	13.25689
255	2R9S	JNK1	133.2811	176.6596
279	2RG5	p38a	86.5844	179.9762
287	2RG6	p38a	86.26518	179.9845
2A6	1H1Q	Cdk2	145.8631	156.2639
2A8	3ZYA	p38a	9.710723	47.80782
2AN	4N3E	Hyp1	8.244885	149.6814
2AN	4A8V	Betv1J	24.47552	40.36507
2AN	3CFN	TTR	25.43333	98.02879
2AN	4A80	Betvia	31.92091	35.82387
2AN	4A86	Betvia	34.71792	37.1747
2AN	3PXQ	Cdk2	37.12462	35.24423
2AN	2WOR	S100A7	51.04948	12.2788
2AN	6AWR	PR10a	67.79221	167.7577
2AN	2ANS	Fabp4	69.76154	158.7841

2AN	3PXF	Cdk2	125.5319	161.6422
2AN	3PXZ	Cdk2	126.6514	162.9114
2AN	1TXC	SPE16	126.8421	169.3256
2AN	5AUT	Dapk1	129.7065	4.383714
2AN	3WBG	Fabp3	146.5812	145.0884
2AN	3PY1	Cdk2	146.7436	128.0431
2AN	1EYN	murA	148.1682	153.7448
2AN	4A81	Betvia	163.3704	62.83123
2AN	1OW4	pheromone	169.7534	156.1308
2AN	4EZ7	Cdk2	176.2159	66.51214
2KC	4NFN	Ttbk1	14.58223	40.63369
2KR	4P1R	Pde10A	172.4742	162.864
2NL	4P72	Phet	25.67623	178.0357
2O5	4NW7	Pde4B	152.841	5.841831
2P5	4NWM	BTK	179.3631	178.8775
2P7	4AN9	MEK1	178.855	47.81572
2UG	4PH4	Pik3C3	173.7896	179.9705
2UY	4OP3	Gckr	6.317519	9.276659
2V1	4OT6	BTK	8.57621	160.9782
2V3	4OTR	BTK	5.83213	174.4925
2V6	4ORM	Pff0160C	22.11968	10.85348
2V6	4OQV	Dhodh	130.701	163.1164
2V6	4ORI	Dhodh	134.535	154.8946
2VL	4OTF	BTK	15.08548	172.905
2VL	5P9F	BTK	16.0624	166.2451
2X6	4PX6	Syk	179.9504	0.001863
31Y	4PMT	TrkA	168.7246	164.3087
32W	4BB4	Ephb4	6.66536	8.048389
349	3ET7	FAK2	172.7858	167.6716
34W	4QMV	Stk24	162.1652	2.629207
34W	6QAS	Ulk1	168.5324	177.0304
34W	5VD2	Wee1	174.5833	2.520803
37O	4QQC	Fgfr4	160.2122	173.4523
37O	4QQ5	Fgfr4	170.8104	158.2737
37Q	4QPS	Jak3	13.97327	17.26507
38K	4QSM	Ldha	143.9004	155.1196
38P	3MVL	p38a	15.70105	73.60014
38Q	4QT0	Ldha	170.4538	112.1778
390	6OPK	ERK2	1.155132	136.8053
390	4QTE	ERK2	162.1227	153.2235
39P	3MVM	p38a	176.9378	86.7583
3BJ	4QRD	mtrna	41.68233	165.2171
3BM	3EQC	MEK1	178.918	50.45421

3D8	4U43	HGK	3.008074	1.061337
3D9	4U44	HGK	177.7001	18.34558
3DC	4U45	HGK	15.38038	0.28029
3EW	4U7Z	MEK1	53.93309	171.4933
3EY	4U81	MEK1	50.45589	174.2605
3FF	3QUE	P38a	179.7488	61.18585
3I6	4BGH	Cdk2	175.568	173.9818
3JZ	3FZR	FAK2	170.7593	154.5483
3LI	3STR	Pdf	109.175	179.578
3NG	3PE1	CK2	0.991529	25.44215
3NG	6P5S	Hipk2	1.036934	36.55684
3NG	6FYV	Clk4	4.152022	41.54809
3NG	6HMB	CK2	5.187097	24.39094
3NG	6FYP	Clk3	7.865513	39.25759
3NG	6FYL	Clk2	9.639897	38.21785
3NG	5O11	Pim1	15.51717	9.305729
3NG	6KHD	Clk1	17.6012	21.09973
3NG	6KHF	Clk3	27.62251	14.81336
3NG	6KHE	Clk2	29.58461	17.92373
3NG	6ISJ	CK2	171.8265	36.09797
3NG	6K3L	CK2	175.2201	34.15618
3NG	3NGA	CK2	179.6201	34.84988
3NV	3NUX	Cdk6	128.8833	0.35577
3O4	4BBE	Jak2	171.8402	168.5947
3OR	3ORN	MEK1	54.72859	166.6437
3OS	3OS3	MEK1	51.22995	174.1247
3OU	4RFY	BTK	6.051902	169.299
3OV	4RFZ	BTK	5.554651	176.2778
3P0	4RG0	BTK	9.650447	169.831
3QS	4RJ8	EGFR	144.4997	25.67396
3QS	4RJ3	Cdk2	176.8569	165.4547
3QW	4RJ4	EGFR	20.67032	148.9959
3QY	4RJ5	EGFR	177.5092	5.703438
3R0	4RJ6	EGFR	7.812114	172.6485
3R1	4RJ7	EGFR	178.7678	9.107449
3TA	3THB	Plk1	12.37616	122.9714
3WK	4X2G	ALK5	57.20694	9.061596
3WN	4X2J	ALK5	59.96948	10.27665
3WO	4X2K	ALK5	59.96948	10.27665
3YT	5UT2	Jak2	0.13018	20.77963
3YT	4RX9	Syk	168.0786	18.0199
3YV	4RX7	Syk	175.0452	11.46275
3YX	4RX8	Syk	170.2216	19.29447

406	2E2B	Abl1	179.9821	92.43672
40M	4XCU	Fgfr4	123.4973	174.1965
447	2VRX	Aurkb	173.9141	135.2898
44C	2FBR	TTR	141.0692	154.3419
481	4OT5	BTK	2.045684	173.2521
4BM	3EQG	MEK1	0.00966	54.57378
4BM	3VVH	MEK1	175.7675	50.61008
4CV	4YFF	Tnni3K	15.89538	171.8795
4CW	4YFI	Tnni3K	7.193287	168.7333
4DF	4YJO	Syk	163.3485	9.606511
4DJ	4YJR	Syk	150.7732	179.2936
4DK	4YJQ	Syk	164.0686	0.62224
4DL	4YJP	Syk	0.094144	163.4464
4DN	4YJS	Syk	163.7059	0.008404
4DO	4YJU	Syk	177.8434	176.3007
4DQ	4YJT	Syk	177.1711	177.8387
4EF	4YHT	Braf	172.6709	3.271303
4FR	3DBE	Plk1	179.8913	179.9137
4GU	4GU9	FAK	18.07641	9.375898
4K4	4YZM	Roco4	2.810363	133.8561
4K4	5VBO	Brd4	170.23	14.40851
4K4	5OQ6	Chk1	170.2953	161.9143
4K4	5OOT	Chk1	170.9899	158.392
4K4	5OQ5	Chk1	172.0626	158.2107
4K4	5WA5	Brd4	175.0929	16.33536
4K5	4YZN	Roco4	3.142298	151.5713
4KT	5AX9	TNIK	171.5387	161.2933
4LH	4Z16	Jak3	173.1949	143.565
4LI	3SVJ	Pdf	103.4895	2.846906
4MG	4RSS	Syk	176.7422	172.7337
4QB	1RWN	CASP1	1.434623	14.81571
4RJ	4ZTR	Aurka	22.12391	153.6003
4RK	4ZTS	Aurka	6.382213	144.3342
4SP	6BSS	Jak2	24.63104	1.266976
4SP	5LQF	Cdk1	152.0464	162.8389
4SP	1H1S	Cdk2	154.607	164.9255
4SP	4EOK	Cdk2	156.9889	160.0503
4SP	2IW9	Cdk2	160.7119	161.3203
4SP	4EOR	Cdk2	161.2969	161.4281
4SP	5M57	Nek2	163.6169	163.0237
4SP	2IW8	Cdk2	165.6836	161.1881
4SP	2C6O	Cdk2	168.0671	163.3601
4T9	4C4E	TTR	140.6811	5.412783

4UQ	5BPY	BTK	179.4311	1.358666
4WE	5BYZ	ERK5	4.95642	13.41832
4WG	5LRQ	Brd4	13.45897	10.49417
4WG	5BYY	ERK5	152.5401	179.586
4YV	5C8K	EGFR	152.9819	16.45501
4YW	5C8M	EGFR	16.75351	155.8097
4YX	5C8N	EGFR	15.93727	149.898
4Z8	5CAL	EGFR	13.86065	149.3871
4ZB	5CAN	EGFR	16.91121	144.7397
4ZG	5CAO	EGFR	19.72158	149.7543
4ZH	5CAP	EGFR	15.23765	152.61
4ZJ	5CAQ	EGFR	22.31094	146.2195
4ZQ	5CAV	EGFR	13.84505	146.6046
4ZQ	5CAS	EGFR	20.35707	146.7968
4ZR	5CAU	EGFR	20.44495	143.1638
50D	5CEO	DLK	173.3447	175.5398
50H	5C26	Syk	169.6681	176.7213
50J	5C27	Syk	1.36995	172.9915
51W	5CI7	Ulk1	1.858748	1.449245
55S	5CYI	Cdk2	154.1725	160.6135
584	5WIK	Jak2	11.61305	15.74459
584	5D9K	Rsk2	127.6854	176.8705
58C	5D7A	TNIK	167.9039	164.3635
58V	5DA3	Ptk6	155.5799	19.44177
59B	5DCZ	Tnks2	179.566	171.1659
59L	4ZOF	Trpd	63.09146	176.4412
5BS	6BRW	Jak2	173.5868	16.29827
5BS	5DH3	Stk3	176.0322	3.923558
5C4	5DL4	Esr1	136.8998	154.3333
5C6	5DKS	Esr1	147.6659	122.7703
5C9	5DKB	Esr1	178.7506	97.0256
5CC	5DK9	Esr1	26.80197	41.48479
5DG	5DMF	Esr1	133.5467	166.2172
5E4	5DQE	Tead2	156.9708	58.31805
5EA	1S9I	MEK2	9.383585	41.45331
5ES	5DP0	Esr1	21.4675	85.93402
5EV	5DT2	Dot1L	147.8702	5.1659
5EW	5DSX	Dot1L	0.834455	176.2786
5FR	3DBF	Plk1	179.8374	0.02
5MS	3EXO	Bace1	111.0499	10.51578
5NW	5EH0	TTR	149.1336	179.6324
5O1	5EHO	TTR	141.5196	175.9448
5O7	5EI2	TTR	145.7379	2.675074

5OE	5EI8	TTR	5.389841	139.438
5OQ	5EI6	TTR	7.274642	132.6497
5Q3	5EM6	EGFR	178.3472	6.588922
5Q4	5EM7	EGFR	110.9217	96.91739
5Q4	5EM8	EGFR	129.3678	57.18805
5QS	5ENN	Pik33	0.113681	172.3219
5T8	5F00	Bace1	39.78552	171.5205
5UU	5WFO	Hras	14.70413	54.31094
5UV	5WFQ	Hras	148.8463	92.90575
5UX	5WFP	Hras	75.597	160.9097
5UY	5F4N	Chk1	15.68416	163.0408
5XJ	5FD2	Braf	77.8178	168.2573
5Y0	4AN3	MEK1	167.2473	52.9809
5Y2	5H8B	CK2	1.014609	174.3616
5Y3	5H8E	CK2	177.1321	177.4701
5Y4	5H8G	CK2	175.2539	3.71899
60B	5HCX	EGFR	14.4402	146.2297
60D	5HCY	EGFR	21.48116	144.4223
60E	5HCZ	EGFR	22.21703	148.3228
61L	4ZOJ	Trpd	69.55467	171.764
62L	4ZOK	Trpd	109.7657	162.3246
633	5HG5	EGFR	178.4776	178.4209
636	4GIU	Trpd	67.01021	173.6231
63N	5HIC	EGFR	20.34304	145.1021
64Q	5HQ5	Brd4	5.32753	118.0312
658	5V03	SK2	4.232065	43.34307
65B	1SV5	HIVrt	165.2647	85.22622
65B	3M8P	HIVrt	169.2985	19.42037
65B	3MEC	HIVrt	178.235	17.69018
65B	3MED	HIVrt	179.1672	22.31323
664	4A4O	Plk1	2.130985	135.391
683	4GKM	Trpd	27.3382	15.09199
685	3EMG	Syk	173.7681	5.88001
69Q	5I96	Idh2	28.97857	156.1449
6AE	5IEY	Cdk2	178.4092	163.7898
6AF	5IEX	Cdk2	175.8361	166.4519
6CA	2FLM	TTR	148.3575	149.3402
6CP	1H1R	Cdk2	146.6737	159.389
6DC	5ITA	Braf	47.88048	19.13265
6GE	5J79	Ripk2	67.35047	175.5761
6H4	5J8I	TAK1	159.1354	172.8466
6HF	5J9L	TAK1	131.8571	3.690453
6J3	5L4I	TTR	3.487743	41.95408

6J3	6GNR	TTR	5.763853	73.42822
6JS	5JEB	EGFR	0.71799	33.07935
6LP	5JMS	Cdpk1	127.0383	78.12089
6O2	5L6H	Uba1	13.48729	28.72394
6P6	5L6O	Ephb3	71.99381	9.003185
6P8	5L6P	Ephb3	70.97248	13.72383
6P9	5JSG	Spin1	5.297966	39.00501
6PD	5JSJ	Spin1	79.94898	127.6214
6PT	5K1I	Pde4D	41.14331	5.736846
6PV	5K00	Melk	156.2198	163.5317
6Q2	5K32	Pde4D	25.42859	25.60104
6Q4	5K3Y	Aurkb	177.0651	9.058443
6T5	5KH9	Hdac6	7.62821	16.93857
6U7	5KKR	Ksr	168.4596	91.10552
6UX	5KNJ	Mlkl	177.2673	177.1545
6XK	5KU8	CK2	165.2284	0.903417
6XL	5KUP	BTK	15.30822	173.6027
6XT	5KWH	CK2	4.287252	0.528791
6Z2	5KZ7	Mark2	149.6739	0.869769
6Z5	5KZ8	Mark2	151.1949	2.442055
6Z7	4D2P	Melk	162.0606	160.0517
6ZV	5L2S	Cdk6	179.0296	148.0534
6ZZ	5L2T	Cdk6	136.7781	2.29827
72L	5NEV	Cdk2	164.4541	158.8972
741	3F5P	Igf1R	37.71059	36.59957
746	3OCS	BTK	1.792014	176.9315
746	4Y93	BTK	3.948205	178.5637
746	4Y95	BTK	4.914937	177.2149
748	5GRN	Pdgfra	172.0449	76.51796
77V	5T68	Syk	11.52417	157.7283
78L	5TBE	p38a	11.39903	40.8609
78Z	5TBO	Pff0160C	24.3933	35.36311
79C	5TA8	Plk1	168.6708	0.151121
79C	5VBP	Brd4	176.2981	170.6913
79D	5TA6	Plk1	179.7484	165.6765
79Q	5TCO	p38a	11.10055	35.91694
7A7	5LXD	Dyrk2	171.1253	88.34982
7AA	5LXC	Dyrk2	173.5461	74.98277
7CE	4C4F	TTR	139.0295	1.37704
7DJ	5TKB	Pde4D	56.93641	13.95069
7DZ	5UU1	Vrk2	138.1397	7.995443
7DZ	5UVF	Vrk1	156.1549	3.006887
7G6	5P9G	BTK	6.144221	173.0144

7G7	5P9H	BTK	175.9126	176.5968
7GG	5M55	Nek2	173.2772	177.4727
7GJ	5M53	Nek2	172.7336	2.326047
7GL	5TKD	Tyk2	39.58342	172.0896
7HK	4FTU	Chk1	151.764	172.0896
7KD	5MAH	Melk	161.5762	1.37589
7KF	5TT7	Syk	8.263773	176.2394
7KW	4C3F	Lck	175.0566	6.562871
7LS	5MEM	Pygm	11.90339	7.548927
7MY	5TX3	Melk	173.901	157.0642
7O3	5MJA	Ephb1	173.7296	74.60794
7P1	5BO2	Trpd	166.3272	145.9977
7P2	5BO3	Trpd	145.9433	156.9741
7P3	5BNE	Trpd	17.31672	29.80694
7PY	2ETM	FAK	175.9974	160.7715
7RO	5AP5	TTR	6.010372	133.6643
7RO	4C4G	TTR	13.20906	124.016
7RO	4C4H	TTR	179.2868	130.8509
7X1	2VWU	Ephb4	8.158563	65.3243
7Z6	5X4Q	Bcl6	121.1599	0.618601
7ZF	5X4M	Bcl6	128.272	0.63731
7ZL	5X4N	Bcl6	131.5652	177.8358
7ZO	5X4P	Bcl6	124.0089	0.35601
7ZV	5U72	Mr1	95.80759	14.93215
80L	5X9P	Bcl6	177.6384	127.8867
80R	5X9O	Bcl6	129.2004	179.0069
855	3HMM	ALK5	26.98858	7.506839
8BM	5UGB	EGFR	7.946457	171.4256
8BM	5UGA	EGFR	178.9004	178.0199
8C5	3DPK	Csf1R	11.51188	142.8438
8E9	4CD1	Entpd2	175.1373	139.4012
8E9	4CD3	Entpd2	178.3674	133.6993
8GN	5N20	Bcl6	56.17448	18.66858
8H0	5XY1	Lyn	4.828405	165.6721
8HN	5N21	Bcl6	177.2641	124.9406
8LU	5Y25	EGFR	40.20307	22.40532
8LY	5USQ	ALK5	42.0271	4.302746
8M4	5UTE	KSHVp	109.3349	10.42661
8MA	5UTN	KSHVp	104.9925	12.49096
8MY	5UT6	Jak2	15.78385	179.3664
8N4	5UV3	KSHVp	104.1251	13.42279
8N4	5V5E	KSHVp	112.1405	14.97757
8NO	5Y5N	Sirt2	10.51301	22.89076

8OF	5Y4Q	Tc00	27.93952	22.93332
8OR	5Y5T	Syk	0.138636	177.1546
8OU	5Y5U	Syk	51.99418	40.752
8OY	5UR3	KSHVp	108.311	10.77757
8OY	5V5D	KSHVp	113.8707	179.6881
8PT	5N7V	TTR	2.137287	138.6087
8Q6	5MME	Cbp	17.19813	104.1298
8QE	5N93	TTR	149.0828	9.13989
8QZ	5NA0	TTR	152.1609	13.91048
8RC	5Y9T	EGFR	176.3172	169.4794
8S3	6AKW	Fto	73.4128	162.989
8TM	5ALR	Ephx2	31.96505	179.665
8X2	5NGU	ERK2	137.6146	6.672327
8X7	5VDK	Wee2	142.495	4.036262
8X7	5VD0	Pkmyt1	163.5788	179.1491
8X7	5V5Y	Wee1	164.8275	171.7287
925	3KXZ	Lck	169.3883	62.80051
92M	3H9F	TTR	12.34489	131.4186
937	2YAC	Plk1	5.430124	125.0523
939	4A4L	Plk1	174.992	144.3402
93J	5VCW	Pkmyt1	31.39856	40.72014
96M	5VC5	Wee1	152.3669	11.13105
97B	3H10	Aurka	159.8278	6.490381
981	5VEE	Pak4	154.9161	4.726516
98D	5VDA	Wee1	176.3987	152.5116
98G	5VD9	Wee1	175.972	155.0299
98M	5VD8	Wee1	179.0609	165.0807
99J	5VD4	Wee1	156.2005	3.468566
99K	5NUD	Jtk2	144.3663	20.45949
99M	5VD5	Wee1	0.691152	155.9535
99V	5VD7	Wee1	3.722382	157.1748
9AJ	5VFI	BTK	11.3589	163.007
9B1	5VGO	BTK	12.91344	169.2627
9BM	4BW3	Brd4	26.35671	70.12357
9BM	6DML	Brd4	141.5503	129.9593
9ES	5VND	Fgfr1	1.245549	115.797
9FC	6JMF	Fes	3.76838	170.7562
9JO	5ZTO	EGFR	9.589904	167.6769
9KO	5ZXB	Tnk2	144.8944	16.20212
9M3	5ZZ4	BTK	4.515893	0.27032
9NH	3B2W	Lck	3.438017	150.4966
9RM	4D5H	FAK	171.5814	9.588034
9TO	6ACR	Acvr1	173.8941	14.13663

9VS	6MEP	Mer	166.4808	143.1061
9W1	5W49	Ahcy	59.70425	165.5405
9XG	5W88	P19429	75.04784	53.54752
9XG	5WCL	P19429	148.6485	152.4971
9YQ	5ONE	Aurka	178.9936	178.4421
9YS	5W85	Irak4	130.775	179.8061
9YV	5W86	Jak3	154.5285	1.621868
9YY	5W84	Irak4	129.9156	0.21375
9ZP	4B6L	Plk3	148.8064	6.624497
A0H	2XNG	Aurka	12.94967	175.4037
A0T	5OP4	Chk1	174.3745	160.2997
A1K	5OP7	Chk1	171.6917	160.989
A5B	5IA0	Epha2	7.376212	165.468
AA2	1OEC	Fgfr2	0.943376	173.7873
ABZ	1S9G	HIVrt	36.38108	58.19816
AD5	5LJJ	TTR	3.354683	152.1598
AD5	2VGO	Aurkb	20.71461	155.9253
ADB	1S9E	HIVrt	2.70811	164.9443
AIZ	2B1P	JNK0	34.60401	33.37585
AM5	3BYS	Lck	168.1151	150.8268
AM6	3BYU	Lck	157.9233	156.5805
AM9	3BYO	Lck	171.4484	160.8391
AP9	2F2C	Cdk6	32.73457	114.9972
AQ1	5WI0	Nampt	10.68406	175.4121
AQ4	6DWN	Cyp1A1	23.44932	170.7697
AQ4	4HJO	EGFR	38.68345	10.47467
AQ4	1M17	EGFR	41.5958	5.072963
AQ6	6IQN	Ntrk1	26.28001	38.37637
AQB	3TZ7	Src	147.689	22.73249
AQG	5WIJ	Jak2	13.64819	8.521518
AQG	6JGM	Cdk2	169.6855	172.7899
AQM	3TZ8	Src	163.1882	86.91597
AQU	3TZ9	Src	159.5538	67.37694
AQZ	2BAK	p38a	11.28003	63.90564
AQZ	4A9Y	p38a	11.28003	63.90564
AS6	2XVD	Ephb4	172.0441	168.1314
ASH	2XNE	Aurka	171.3201	8.686182
ASW	4G0U	Top2B	30.28019	38.30746
AU5	5AP3	TTR	179.4089	145.5373
AU5	5AP0	TTR	179.7436	136.7131
AVD	3CEM	Pygl	177.0575	81.19579
AYS	5Q1Z	Dclre1A	19.19778	35.56798
B1A	5Q28	Dclre1A	0.195073	44.75498

B1E	4RZW	Braf	6.900045	65.85226
B1E	4G9R	Braf	10.06017	66.29025
B1E	5HID	Braf	11.84338	61.20786
B5S	4UMQ	Melk	157.0407	164.2685
B5U	4B6C	Gyrb	12.31612	146.2934
B7B	6EIP	Dyrk1A	169.254	125.7903
B90	3KF4	Abl1	2.843574	6.123673
B9K	6EKD	JNK3	135.0727	171.412
BBM	1S9J	MEK1	65.68671	165.1343
BGE	6EMH	JNK3	141.1548	173.5156
BII	2JKM	FAK	150.2731	3.526853
BJG	4D1S	Jak2	3.25141	0.38501
BNB	6EP9	BTK	178.5316	177.1161
BNY	6AP8	D14	164.944	145.7963
BNY	6AP7	Dad2	174.3631	150.9062
BPG	1QHI	UL23	176.2	23.037
BRA	3CE5	Tele	20.70953	43.57183
BT9	4DBU	Akr1C3	48.93631	19.22323
BTH	1RWO	CASP1	1.511048	18.44008
BX7	4JL9	Tbk1	157.7736	0.386777
BX7	4IM2	Tbk1	163.6907	179.708
BX7	4IM3	Tbk1	164.6783	179.7055
BX7	4EUU	Tbk1	171.4639	4.06997
BX7	4IWP	Tbk1	174.4281	155.4478
BX7	6O8C	Tbk1	175.2536	174.2623
BX7	4EUT	Tbk1	175.6515	1.114315
BX7	6O8B	Tbk1	177.8549	168.9753
BX7	4IW0	Tbk1	178.9535	2.843324
BXM	6AUB	BTK	3.842178	169.0487
C0H	6EW6	Bcl6	134.837	172.0644
C5N	5MRB	TTR	134.0818	4.857967
C5N	5O91	TTR	135.5494	174.5476
C6F	6JQR	Flt3	169.6438	10.18647
C87	6FDZ	Ulk3	3.46801	154.4838
CDK	2XMY	Cdk2	160.7942	170.7166
CF1	3HP9	Sbcb	139.0309	172.7784
CG4	4PV0	Syk	177.9493	1.48926
CG9	4PUZ	Syk	178.5556	0.97809
CJZ	3M1O	TTR	7.07581	43.95251
CK4	2C5V	Cdk2	3.266745	173.6758
CK4	1PXL	Cdk2	179.4459	0.439703
CK5	1PXM	Cdk2	0.88329	165.7187
CK6	1PXN	Cdk2	179.3094	178.9977

CK7	2WEV	Cdk2	150.7476	18.01523
CK7	1PXO	Cdk2	155.7344	19.34865
CK7	4B9D	Nek1	174.5631	161.5587
CK8	2C5N	Cdk2	1.0682	1.6355
CK8	1PXP	Cdk2	179.0857	178.4638
CQ3	4ZZO	ERK2	132.9017	171.5593
CQ7	6B4W	TTR	174.3348	156.0575
CQ8	4ZZN	ERK2	0.117886	131.4642
CQA	4FGZ	Pmt	12.42874	61.42449
CQA	2AOU	HNMT	156.4023	71.82066
CQA	4MWZ	Pvx	179.0732	34.22432
CQQ	3V6R	JNK3	174.4102	151.4619
CQU	3CQU	Akt1	158.4444	11.38913
CT7	1Y8Y	Cdk2	147.7567	153.482
CT9	1Y91	Cdk2	165.5655	139.9001
CVE	6F6R	Casp1	23.00188	2.342499
CVK	6F76	Kras	174.5281	99.06767
CVN	6F78	Akr1C3	178.9003	68.70306
CVQ	6F7B	Bub1	11.65246	168.1481
D0A	6B8Y	ALK5	9.666127	29.06447
D0H	6F94	Acrb	22.14761	176.4283
D0K	6F96	Gyrb	59.8293	171.6525
D0V	6BAG	Ldha	169.3414	164.2743
D0Y	6BAD	Ldha	13.75306	27.44036
D1W	6FA4	Kras	175.1751	150.782
D1Z	6FA3	Kras	175.0509	172.8319
D36	3R21	Aurka	3.677462	165.7132
D37	3R22	Aurka	163.272	158.9609
D47	6BAZ	Ldha	5.475571	65.43916
D4A	6BB3	Ldha	163.1804	163.7967
D65	5BOO	Pff0160C	40.5943	169.5462
D65	4RX0	Pff0160C	41.15683	176.3211
D67	3SFK	Pff0160C	33.368	27.10173
D6I	4BZD	Cdk2	3.884343	164.5895
D9H	3MMF	Ca2	158.6942	1.104096
DB8	5AJQ	Stk10	45.4493	21.43398
DB8	4MXX	Src	47.86824	27.08685
DB8	6OP9	Erbb3	49.48612	25.41777
DB8	5I9X	Epha2	50.79453	30.1259
DB8	4MXO	Src	51.58279	27.24269
DB8	5VCY	Pkmyt1	52.7528	26.2745
DB8	3UE4	Abl1	53.85141	23.06673
DB8	4QMN	Stk24	56.20375	27.2606

DB8	3SOA	Camk2A	58.84815	141.2605
DB8	4MXY	Src	64.5612	0.853205
DB8	4MXZ	Src	64.5612	0.853205
DB8	5VC3	Wee1	66.95996	179.7474
DB8	6FDY	Ulk3	80.99937	168.0382
DIF	4ZBQ	Alb	48.79318	8.768447
DIF	6HN0	Alb	52.77099	11.98303
DIF	1DVX	TTR	58.00292	18.94999
DIF	6HN1	Alb	68.92918	13.49811
DIF	4OJ4	Pparg	76.09675	179.7902
DIF	4UBS	Cyp28	77.90962	10.11686
DIF	5U1R	Mr1	79.54752	41.81148
DIF	2WEK	Zadh2	81.34079	3.626742
DIF	3N8Y	Ptgs1	104.2092	179.0574
DIF	4XTA	Pparg	107.9089	163.8319
DIF	5DBY	Alb	111.4479	2.425278
DIF	1PXX	Cox2	115.4407	171.1713
DIF	4ZBR	Alb	116.0015	169.7809
DIF	4Z69	Alb	120.2106	117.4517
DIF	1NR6	Cyp2c5	120.6352	150.2994
DIF	3CFQ	TTR	124.5603	4.347992
DIF	3IB0	Ltf	127.4583	163.7545
DIF	2B17	PLA2	137.1205	0.935439
DIF	1SV9	PLA2	137.7709	0.594725
DJK	2HWP	Src	0.514334	179.2846
DJK	3LOK	Src	0.973499	31.67752
DJK	4LQM	EGFR	10.78383	35.30507
DJK	2J5F	EGFR	14.37925	32.30448
DT1	2C6I	Cdk2	152.4389	153.7609
DT2	2C6K	Cdk2	145.4724	157.2127
DT4	2C6L	Cdk2	141.5092	154.963
DT5	2C6M	Cdk2	142.6337	160.3278
DT5	2C6T	Cdk2	142.7918	158.8764
DTJ	6BIK	BTK	2.394365	169.9388
DTQ	5AMN	Ret	9.041635	36.5332
DTQ	3NYV	Cdpk1	23.54222	42.76113
DTQ	6U0K	Ttbk2	175.5128	156.5107
DTQ	4BTK	Ttbk1	178.3615	145.6284
DTQ	1DI8	Cdk2	179.7293	0.01515
DWF	4MXC	Met	165.0562	0.976159
DWG	6BL1	Idh1	138.1595	173.7251
DWH	3MNA	Ca2	153.5152	4.24182
DWP	6BKX	Idh1	137.5193	1.259566

DWS	6BL2	Idh1	136.005	0.76303
DWT	6FNG	Epha2	76.65183	16.53635
DWT	6FNJ	Ephb4	77.74329	14.19476
DXH	6FNF	Epha2	94.72751	179.9443
DXH	6FNI	Ephb4	95.71368	6.817232
DY7	6BM7	Tb927	29.54056	131.947
DYK	4D2R	Igf1R	160.9364	157.3291
DYK	4D2S	TTR	179.627	141.872
DZO	3E7V	Haspin	8.095396	171.1935
E1B	3PE2	CK2	3.090842	24.92907
E1D	6BP0	Vrk1	162.9978	13.37541
E5M	6BRU	Vrk1	160.5638	5.091683
E70	3HKC	ABT751	152.1836	4.966553
E7W	6FUI	Cfd	4.638333	31.41375
E86	3I60	ERK2	177.3374	138.072
E88	6FUH	Cfd	6.384103	20.96309
E8D	6BTW	Vrk1	165.9914	13.10458
E94	4FV7	ERK2	168.5951	156.9488
E9Z	3QBN	Aurka	29.8049	152.7518
EA7	6CFM	Vrk1	155.7591	15.35987
EDJ	6BX6	Prkaa2	13.44114	149.273
EK7	4FV4	ERK2	169.5679	161.9
EKT	6G3C	Jak2	168.422	15.52362
EQ7	6C8C	Q920Q2	161.6011	5.010314
EUI	4LMN	MEK1	41.73065	5.747362
EUI	4AN2	MEK1	53.08733	176.8839
EX1	6CD4	Brd4	20.08913	165.9412
F0H	6GJB	ERK2	151.5801	173.142
F0V	6CHQ	Coad	62.30516	13.09888
F1S	4F1S	Pik3g	153.4736	165.7582
F1T	6GJG	Pff0160C	13.77352	21.3766
F2S	6CJ1	Brd4	38.33109	159.012
F3J	6CIY	Brd4	33.80373	156.6438
F46	4L8M	p38a	29.55442	11.28068
F4A	6CJE	Mnk2	34.14347	11.81628
F4J	6CJY	Mnk2	174.7066	12.46004
F62	5GMP	EGFR	2.539508	151.6491
F67	6CK3	Mnk2	5.588961	8.151454
F6M	5X02	Flt3	4.045782	178.9145
F7D	6CMM	Vrk1	22.94592	152.0075
F87	6CNX	Vrk1	8.217336	137.1087
F8E	4BTM	Ttbk1	150.0153	179.2614
F8H	6GQM	Kit	167.407	61.15014

F8K	6GQX	Kras	168.5163	51.79279
F8Q	6GQY	Kras	164.0676	31.27353
F8T	6GQW	Kras	128.1459	67.43283
F8Y	6CQH	Vrk1	136.7357	15.84116
F97	6CQF	HPK1	172.8773	18.47244
F9N	6GT1	Nek7	121.8785	28.9327
F9N	6H0O	Nek2	165.592	168.7807
FB8	6GU7	Cdk1	160.3685	149.4026
FB8	6GUE	Cdk2	170.009	163.4809
FB8	6GUH	Cdk2	170.4106	157.7874
FB8	6GU3	Cdk1	170.5921	166.2242
FBY	6CSW	Vrk1	156.8969	33.13031
FC8	6GU4	Cdk1	8.013367	131.3944
FC8	6GUK	Cdk2	177.4664	135.8086
FCS	6CNX	Vrk1	18.93012	170.3108
FFS	6CUO	Hras	178.3602	171.4231
FFV	6CUP	Hras	11.05067	175.6963
FFY	6CUR	Hras	6.351459	173.7875
FI3	4R5S	EGFR	9.029923	135.7581
FI3	4R6V	Fgfr4	164.298	137.671
FJI	5MTX	p38a	1.146411	42.44405
FJY	4FJY	Pik3g		
FLF	1S2C	Akr1C3	6.277864	71.24699
FLF	3OZL	TTR	13.88797	27.72049
FLF	1BM7	TTR	14.29868	31.70622
FLF	4I5X	Akr1B10	111.1016	179.9858
FLF	5DQ8	Tead2	162.7569	62.99083
FLF	5IKV	Cox2	170.7188	147.2859
FLF	2PIX	AR	174.3882	179.143
FLJ	6CZ3	Ptk6	2.228483	164.2979
FM7	3ZHZ	Dxr	152.4875	146.1152
FMM	1XKK	EGFR	8.568022	55.02535
FMW	6H3K	TTR	137.4345	179.7514
FNA	3S0B	OBP14	7.926195	77.58334
FND	6CJ1	Brd4	43.65189	152.2509
FOV	6D0B	Epas1	0.37032	63.38289
FPX	3TUD	Syk	178.2252	160.3525
FQ7	6D0W	TTR	0.373978	49.94645
FRT	2W05	Cdk2	140.948	3.549175
FRV	2W06	Cdk2	0.764042	158.8341
FS8	3UVQ	p38a	34.22942	7.296819
FSS	3FSF	p38a	176.9348	43.39121
FTA	1PZP	TEM-1bla	177.2125	132.6382

FU9	3R0T	CK2	0.1168	0.10406
FZJ	6CKI	Mnk2	170.204	6.644028
FZP	6D8E	EGFR	170.794	172.0556
FZW	6HEY	Epha2	81.93785	16.18341
G02	6HEX	Epha2	75.10523	20.08198
G0E	6HEW	Epha2	82.37611	11.85862
G0H	6HES	Epha2	84.37322	12.39964
G0K	6HEV	Epha2	72.44472	22.15507
G0N	6HEU	Epha2	86.54349	15.05142
G0Q	6HET	Epha2	80.93086	15.23274
G3Z	5MMP	Gyrb	163.0524	136.1212
G5D	6DBK	Tyk2	1.235307	7.980904
G6J	6DD4	Vrk1	157.6832	13.33034
GJJ	6DI9	BTK	2.796581	175.7697
GK1	2ZAZ	p38a	176.386	85.17893
GMQ	6HRP	BTK	7.231577	176.5297
GMW	6HRT	BTK	176.3092	4.815039
GRY	5QEG	Ptpn1	9.265914	38.07499
GRY	5QHX	Parp14	18.9322	52.63738
GV2	6HVH	Pfkfb3	156.0709	141.7458
GV5	6HVI	Pfkfb3	144.0246	154.739
GV8	6HVJ	Pfkfb3	146.5978	151.7808
GW7	2R4B	Erbb4	50.46183	3.485661
GW8	4AOT	Stk10	3.137024	151.9517
H0S	5QGK	Nudt7	24.98799	2.621356
H82	6I8Z	FAK	11.1988	145.0807
H8H	5VD3	Pkmyt1	1.441634	52.50111
H8H	5VCX	Pkmyt1	1.948584	51.02538
H8H	2H8H	Src	3.458397	50.15534
H8H	4QMX	Stk24	11.11398	63.38451
H9Z	6IBX	Pfkfb3	161.7501	136.9591
HAK	6IC0	Pfkfb3	139.8218	160.2612
HAT	6IBY	Pfkfb3	175.1417	128.6154
HAW	6IBZ	Pfkfb3	143.2656	147.8524
HB9	5MTY	p38a	45.43686	2.596454
HDT	1OIT	Cdk2	165.6422	172.2777
HDY	1OIR	Cdk2	6.265492	138.4913
HET	2BDJ	Src	1.911947	0.249101
HG5	6Q3Y	Brd4	16.62451	1.1375
HG8	6Q3Z	Brd4	12.00177	19.04006
HHB	6Q4J	Cdk2	151.6443	2.709527
HKI	3W2Q	EGFR	29.01159	33.82901
HKI	2JIV	EGFR	39.88442	32.68717

HMW	6Q7E	Epha2	76.8152	17.50887
HNZ	6Q7F	Epha2	49.66869	7.73396
HO5	6Q7B	Epha2	77.79514	16.16923
HO8	6Q7G	Epha2	75.15054	17.54398
HOK	6Q7C	Epha2	72.17969	21.05499
HOT	6Q7D	Epha2	75.92588	19.67189
HPM	2C6E	Aurka	171.9076	144.7386
HUY	6E5G	Tead2	42.02729	68.33756
HVH	6QAV	Ulk2	168.0298	162.0735
HYZ	2RGP	EGFR	58.38881	3.479968
I0D	5MKY	Brd9	111.7547	168.6513
I19	2W17	Cdk2	148.5196	178.139
I1P	1URW	Cdk2	1.631028	158.5117
IAQ	3T9T	Itk	0.950893	76.1526
IB1	5K14	HIVrt	168.4778	166.7046
IBI	5V67	Brd4	7.334729	1.917856
IBI	5VBR	Brdt	156.7376	23.82173
IBI	3FC2	Plk1	172.0305	177.1255
ID8	3R43	Akr1C3	0.142726	87.80946
ID8	4G2Z	Ltf	131.4211	5.771579
ID8	4JQA	Akr1C2	147.6299	167.018
ID8	5IKR	Cox2	159.3182	156.2428
ID8	2XN3	Serpina7	168.8412	76.41615
IHJ	1U9X	CTSK	168.857	48.8285
IHZ	3DKO	Epha7	120.1115	6.137896
IM9	2VV9	Cdk2	165.4428	171.5817
IPV	5KBQ	Pak1	175.6523	179.4277
ITI	3LZB	EGFR	23.41202	4.303109
IXH	3BEA	Tie2	168.3008	5.931412
IZG	3PP1	MEK1	44.71609	3.389368
J2T	4BO7	Fabg	176.731	3.865763
J43	3MHO	Ca2	38.9348	6.961356
J45	3M40	Ca2	168.5709	171.0513
J4X	4CRG	F11	162.6531	167.8143
J4Z	3I68	Pff0160C	33.90039	20.01725
J5Z	3I6R	Pff0160C	55.71384	169.4733
J9D	6M9H	Jak2	15.70167	0.827195
J9G	6M9L	p38a	85.50998	159.6953
JHV	5QEB	Ptpn1	37.7898	99.30019
JIN	2HZI	Abl1	155.2341	9.303608
JJM	5QEI	Ptpn1	169.2401	147.8252
JJM	5QJL	Nudt5	171.0538	167.7231
JK2	3FI3	JNK2	45.66372	18.08324

JL2	6QY9	CK2	163.1515	173.7088
JLJ	4KO0	HIVrt	23.72141	4.107313
JMS	5IKQ	Cox2	0.211545	123.0227
JMS	4N6P	Ltf	113.8822	58.2495
JMS	4QKN	Fto	148.0285	103.7259
JMS	6IJX	Akr1C1	170.6377	62.24931
JMS	3R6I	Akr1C3	177.6676	101.3993
JNK	2EXC	JNK3	8.577719	47.24266
JQP	6MH1	Brd4	129.4093	173.0839
JQY	6MH7	Brd4	134.5615	171.7739
JWF	4BTY	Aoc3	108.8964	164.7293
JZ8	6E0B	Pff0160C	38.3899	16.9304
JZ8	3I65	Pff0160C	40.02838	9.933116
K9T	6RN8	Ripk2	10.76113	56.68278
KA2	6RNA	Ripk2	6.252894	62.99329
KE7	6N6O	TTR	173.635	140.033
KIM	3CJG	VEGFR2	171.3383	165.6328
KLM	6NFH	BTK	160.0409	8.365056
KR4	6NJI	Pde4D	141.5132	23.77338
KRD	6NJH	Pde4D	26.53413	135.8397
KSF	2ZM1	Lck	106.5374	166.2436
KSL	2ZYB	Lck	99.73444	163.3622
KSM	2ZM4	Lck	99.85689	6.425442
KWJ	6NPN	Vrk1	153.2869	18.60612
KZI	3AC1	Lck	176.8181	6.019477
KZJ	6NSL	Tyk2	34.06073	175.6033
KZL	3AC4	Lck	164.4611	28.45989
KZM	3AC5	Lck	175.4387	0.331087
KZP	6NSQ	Braf	29.73981	37.50408
L51	3HV4	p38a	24.7893	57.30008
L8V	3L8V	Met	179.8778	18.27493
L9A	6NZH	Tyk2	145.937	3.50658
LB7	6NZP	Tyk2	148.3522	1.1131
LCJ	6Q0J	Braf	64.98808	158.9087
LCJ	6Q0T	Braf	65.00614	158.9276
LCJ	6PP9	Braf	65.03559	158.9501
LCJ	6NYB	Braf	65.93251	167.8211
LD9	6PMF	Dsba	28.95564	44.50039
LHL	3PJ1	BTK	7.841922	147.1936
LHL	3KMM	Lck	169.7786	175.514
LI8	1Z5M	PDK1	5.546006	156.8521
LJF	3O51	Aurka	165.5376	144.463
LQQ	2EUF	Cdk6	4.533333	163.8246

LQQ	5L2I	Cdk6	4.876072	109.5644
LUG	3EQB	MEK1	53.34832	172.3135
LUR	4IK6	TTR	32.36665	159.3131
LUR	4IIZ	TTR	140.0109	66.81821
LUR	4RRX	Cox2 V89W	171.0623	112.4842
LUR	4OTY	Ptgs2	171.2971	113.3592
LUR	4RRW	Cox2	173.722	115.6716
LUR	4RRZ	Cox2 H90W	173.722	115.6716
LZ4	2VTJ	Cdk2	1.748743	34.07244
M3K	4ARK	MEK1	177.8322	42.65372
M4M	3VI5	Hpgds	29.23264	124.3756
M8Z	6T29	Camk1D	6.295631	160.979
M92	6T28	Camk1D	171.6014	171.301
M9T	6T3B	Pik3g	115.5099	7.339836
M9T	6T2W	Csf1R	133.1898	8.848208
MBW	6T3C	Pik3g	120.7164	16.6983
MEK	3DV3	MEK1	178.2383	44.8915
MFP	1H07	Cdk2	179.4769	146.2058
MFQ	1H07	Cdk2	179.4769	146.2058
MH4	2XCK	PDK1	7.624732	145.6631
MK2	3KC3	MK2	165.872	177.224
MK3	3KA0	MK2	157.6645	30.57649
MMW	6T6F	Camk1D	173.8386	172.9783
MPZ	1Y57	Src	172.8819	3.597743
MRA	2P55	MEK1	179.6801	51.87988
MSQ	1DI9	p38a	19.6851	59.44399
MT4	3EFK	Met	164.0302	129.5073
MUH	2OSC	Tie2	93.94493	174.62
MWB	3FSI	HIVrt	0.501579	0.94771
MWB	4OCD	DNA	169.1348	157.2613
MZO	3DQ0	Ckx1	157.9027	22.36713
N20	1OI9	Cdk2	158.1124	158.7878
N3F	3QUD	P38a	24.03981	40.05878
N41	1OIY	Cdk2	155.4376	166.1168
N42	5J87	BTK	153.0452	176.2012
N45	6CK6	Mnk2	163.648	11.5707
N4D	3L8X	p38a	73.12379	8.224191
N4W	6TE6	Dot1L	31.6961	2.703346
N4Z	6TEL	Dot1L	38.00884	0.668356
N5K	6TEN	Dot1L	36.89888	1.54489
N66	5N87	TTR	134.1347	8.181383
N76	1OIU	Cdk2	144.92	169.8295

N9G	6OVA	Tyk2	5.447921	14.23204
NFL	2WM3	Nmral1	12.18122	157.7859
NFL	1TD7	PLA2	179.8844	0.4626
NIL	5MO4	Abl1	53.65454	14.58746
NIL	3GP0	p38b	58.72078	19.28444
NIL	3CS9	Abl1	86.88037	7.640295
NJD	4NJD	Pak4	6.918685	3.805711
NK0	3S4Q	p38a	2.614646	73.66734
NU5	2G9X	Cdk2	179.8558	179.4316
NU6	5M51	Nek2	6.484677	171.3156
NVW	3W5E	Pde4B	21.97482	120.0438
NZF	4FNZ	Alk	160.3915	1.050729
NZS	3NZS	Pik3g	133.6531	2.64794
O17	3WYY	TTR	150.0175	17.80568
O19	4BBF	Jak2	4.313705	164.2026
O21	6P69	Fgfr1	24.89807	117.7411
O22	3VQU	TTR	160.6884	7.006861
O38	5AP4	TTR	153.1437	11.47736
O38	5AP1	TTR	163.6542	179.6853
O38	3WYX	TTR	165.6295	4.112611
OFL	1DVZ	TTR	49.31556	3.129759
OQB	1RWW	CASP1	0.286264	0.09661
OVJ	6POQ	Dsba	176.2623	26.93973
P01	2Z7S	RSK1	0.799179	18.4362
P01	2IZU	CSNK1G3	42.68436	10.37845
P01	1YOM	Src	177.7748	6.956821
P16	2FO0	Abl1	155.3841	2.539005
P16	1OPL	Abl1	160.5337	2.671933
P16	1OPK	Abl1	160.6611	2.55156
P16	2G2H	Abl1	168.074	1.147035
P17	5IA3	Epha2	157.6081	7.206569
P17	1M52	Abl1	166.5607	2.511783
P1E	3H3C	Pyk2	174.2432	167.0177
P29	2PVM	CK2	173.5104	170.1633
P2B	3P2B	Pik3g	179.6367	161.2808
P2C	1U21	TTR	7.435629	51.52644
P37	3GFE	p38a	7.057106	79.25597
P38	3BV2	p38a	74.50151	171.9185
P39	3BV3	p38a	77.39272	173.4684
P44	2PVJ	CK2	8.63776	146.4117
P45	2PVK	CK2	8.716384	145.5804
P48	5VD1	Pkmyt1	11.11188	160.2963
P48	5VC6	Wee1	11.68104	155.9132

P48	2WIH	Cdk2	178.7655	142.434
P49	2WIP	Cdk2	179.944	144.0448
P55	2PVL	CK2	9.999188	136.3143
P5C	3FQE	Syk	177.6769	15.27044
P66	3ITZ	p38a	1.698346	79.08594
PDY	3A2C	MK2	36.97612	31.70221
PDY	3WBL	Cdk2	156.793	134.6775
PFE	1KZ8	FBP1	31.27577	14.5105
PO5	3EID	Cdk2	154.571	149.858
POX	3BEL	EGFR	64.65521	5.151825
PQC	3QGW	Itk	9.752742	153.6323
PQC	3QGY	Itk	178.8108	168.7165
PRC	1FPU	Abl1	173.2762	98.5221
PVB	1V0P	CRK2	11.17237	132.4513
PVB	2X7G	Srpk2	15.47711	10.70387
PVB	6BL8	Abl1	170.9269	29.88574
PWU	5AP6	TTR	179.3162	150.5179
PWU	5AP2	TTR	179.6851	154.202
Q1S	6U8L	Wdr5	67.04822	8.674985
QAG	6UL5	HIVrt	8.793473	0.16955
QIG	3GXL	ALK5	19.35872	13.58261
QL2	3I06	cruzain	177.2567	161.5124
QPC	3WD9	Pde4B	14.99841	127.7106
QQ2	2IW6	Cdk2	167.8952	151.5028
QV8	3QV7	Pyk	167.287	160.9536
R0N	5IEV	Cdk2	169.6338	174.732
R24	3HV5	p38a	16.10594	51.14416
R2C	2PRL	Dhodh	0.296812	134.9055
R48	3GCU	p38a	17.22468	50.53905
R49	3HV3	p38a	86.30796	168.1582
R4L	4B99	ERK5	7.979285	129.4938
R4L	6CD5	Brd4	144.5697	29.90777
R6D	6VOV	Syk	0.58231	176.7809
R78	2RKU	Plk1	0.29034	163.8681
R78	6BQQ	Camkk2	0.84987	168.7556
R78	6MF9	Cgd4	12.1869	8.33883
R78	5TCM	Ldbpk	15.28852	140.4059
R78	5VBQ	Brdt	20.57203	158.2378
R78	4O74	Brd4	172.1127	179.6001
R78	4OGI	Brd4	176.3454	175.0141
R78	4I5M	Plk2	178.3999	164.4763
RAJ	3BE2	VEGFR2	82.51015	6.881017
RAJ	2OO8	Tie2	93.88959	177.0813

RBS	2HWO	Src	179.3691	1.495598
RC0	5MW6	Bcl6	168.9322	126.3479
RGJ	5ANV	Nudt1	139.2189	146.0892
RLM	4BN7	Ytjd	123.1474	66.37819
RR1	1I3U	LLAMA	169.6158	177.0741
RYA	6VZH	Vrk1	162.973	9.830188
S19	3SRV	Syk	8.753872	174.2705
S5B	4BW1	Brd4	27.17649	72.39766
S5B	4AKN	Brd2	133.5461	146.7597
SD1	3MNR	Hsp90A	146.7869	138.7074
SK4	3FRG	Pde4B	47.48558	23.41666
SR2	2QQ7	Src	39.64918	8.621628
SR2	2QLQ	Src	57.83157	14.47341
SS6	3GCV	p38a	18.64302	51.76134
ST8	1OGU	Cdk2	154.9935	164.9106
STI	2PL0	Lck	0.591997	102.6275
STI	6NPV	Abl1	1.094504	94.71627
STI	6NPU	Abl1	1.475137	93.50385
STI	6NPE	Abl1	1.628794	91.6466
STI	1XBB	SYK	2.33547	178.1854
STI	4CSV	tkas	2.847781	103.5766
STI	3PYY	Abl1	3.16254	91.27198
STI	3FW1	Nqo2	5.663049	146.7784
STI	6KTN	Pparg	167.2171	0.49717
STI	3HEC	p38a	167.6845	79.62574
STI	2HYY	Abl1	169.8203	103.9709
STI	1T46	Kit	170.1128	82.30795
STI	3MSS	Abl1	171.5766	97.71212
STI	3GVU	Abl2	172.9487	96.96771
STI	6HD4	Abl1	176.0726	96.93327
STI	3MS9	Abl1	176.1037	92.79446
STI	3OEZ	Src	176.2163	100.0497
STI	3K5V	Abl1	176.2485	96.81149
STI	4R7I	Csf1R	178.2702	82.21579
STI	1OPJ	Abl1	179.075	94.03051
STI	2OIQ	Src	179.4694	95.32493
STI	1IEP	Abl1	179.5089	97.44505
STI	6HD6	Abl1	179.6727	93.34138
STI	4BKJ	DDR1	179.6808	95.65299
STI	5MQT	Dck	179.9664	52.88901
STJ	3K5V	Abl1	170.4113	3.248266
SVE	2X9E	TTR	0.24426	127.0866
SVE	5NTT	TTR	157.9751	152.837

SVE	5AP7	TTR	162.7549	163.5832
T2A	3EOC	Cdk2	176.8099	144.0153
T3C	4BCP	Cdk2	149.5647	24.58785
T3C	4BCG	Cdk9	160.9352	21.46995
T3E	4BCK	Cdk2	118.8889	41.91006
T3E	4BCI	Cdk9	171.5828	165.8588
T5J	5ALJ	Ephx2	29.14462	163.685
T6Q	4BCO	Cdk2	149.1445	174.4644
T6Q	4BCF	Cdk9	165.6247	14.89708
T7Z	4BCM	Cdk2	144.2999	167.4509
T7Z	4BCH	Cdk9	162.5375	13.92904
T95	3EQP	Ack1	9.351801	154.0301
T9N	4BCN	Cdk2	146.1909	2.898708
T9N	4BCJ	Cdk9	154.0606	29.62756
TCC	1NHW	FabI	179.6797	95.62315
TJ3	5MWD	Bcl6	0.06628	122.952
TJF	4BCQ	Cdk2	151.068	179.2074
TLF	4G77	Ltf	1.38264	10.65284
TLF	6AP6	Dad2	149.364	175.6397
TLF	5IKT	Cox2	149.9736	159.7723
U52	5MW2	Bcl6	114.4112	17.1994
U55	1JSV	Cdk2	155.5061	166.6679
U98	4BO3	Fabg	13.1765	178.6976
UI1	1SQA	PLAU	117.0209	174.3979
UI2	1SQO	PLAU	124.1576	172.5031
UT0	5MMG	Cbp	16.95159	102.0432
UTH	4BW2	Brd4	22.65035	74.04495
UWN	4FL6	L3Mbtl3	132.5619	159.8747
V04	3V04	MEK1	49.87909	171.6383
VRA	3E8N	MEK1	11.15585	50.18588
VYI	5APH	Rorc	52.35919	10.33853
W19	3W33	EGFR	58.41272	4.165368
W2P	3W2P	EGFR	60.21602	6.659462
W2R	3W2R	EGFR	42.67239	4.014052
W2R	3W2S	EGFR	76.52502	1.427423
W32	3W32	EGFR	53.65923	3.66355
WAI	1Y2F	ZipA	176.4289	157.9005
WCU	5MYW	Ethr	17.52577	46.4372
WI4	4BO5	Fabg	2.819857	7.221155
WY1	5HA7	Akr1B1	112.0615	4.619487
WY1	4BCR	Ppara	132.1323	147.8037
X0A	3QQH	Cdk2	173.4494	44.87848
X20	4C4I	TTR	175.6655	139.9735

X21	4C4J	TTR	173.8926	138.1636
X26	6CIS	Brd4	150.7051	25.7419
X27	6CJ2	Brd4	37.69579	166.0387
X30	5W55	Brd4	20.72519	170.8483
X3G	4XG3	Syk	11.35038	164.7282
X4G	4XG4	Syk	177.3265	172.9934
X5G	5GHV	Syk	164.7103	8.235409
X6G	4XG6	Syk	36.10895	127.7211
X7G	4XG7	Syk	174.3377	177.8308
X8G	4XG8	Syk	172.5008	5.503408
X9G	4XG9	Syk	168.717	9.080475
XL5	6NVJ	Fgfr4	179.508	121.8887
XL6	6NVH	Fgfr4	5.242312	114.4481
XL6	6NVL	Fgfr1	17.0697	125.3012
XL7	6NVI	Fgfr4	0.0885	113.2256
XL8	6NVG	Fgfr4	5.911088	112.5582
XNA	3QA2	Khk	43.08803	165.4
XNB	3Q92	Khk	23.43892	174.0338
XNM	3RZF	Ikbkb	104.0805	82.44788
XNN	3QAI	Khk	29.93575	172.8816
XR1	4EQC	Pak1	172.6777	145.0901
XU2	4JAI	Aurka	139.9208	126.8479
XYW	5AR8	Ripk2	178.7856	178.4325
XZ1	6KO9	RamR	176.7293	154.9575
XZN	5VCZ	Pkmyt1	38.52953	36.61358
XZN	5VC4	Wee1	41.68112	26.66566
XZN	4BC6	Stk10	43.45701	37.90337
YAM	3BZ3	FAK	15.68442	148.7199
YAM	5TOB	FAK2	167.497	157.2944
YAM	5X4O	Bcl6	176.07	132.9936
YM7	2YM7	Chk1	13.78437	157.7403
YM8	2YM8	Chk1	179.9427	176.4703
YQY	4ANB	MEK1	46.34592	179.768
YUN	4LL0	EGFR	19.24979	14.88662
YUN	4LRM	EGFR	31.01638	28.93897
YY3	4ZAU	EGFR	164.7114	169.2208
YY4	6C1B	Fgfr1	2.837739	170.769
Z48	3I5Z	ERK2	6.386152	141.7105
Z48	3I4B	Gsk3B	6.943205	12.06762
Z72	3LY2	Pde4B	10.8846	66.90952
ZD6	2IVU	Ret	8.492786	59.60759
ZTX	3ZTX	Aurkb	15.56836	166.5475
ZUQ	5FRI	ALK5	9.732332	47.92571

ZZ0	2WMD	Nmral1	8.818293	157.6355
ZZF	2WOU	ALK5	155.0633	154.2865
ZZG	2WOT	ALK5	157.9229	152.8338
ZZL	5IA1	Epha2	0.00029	163.5205
ZZL	2WTW	Aurka	4.5152	171.4665
ZZL	2X81	Aurka	151.4953	13.50702
ZZL	2WTV	Aurka	153.1229	11.15527
